# Lgals3 Promotes Calcium Oxalate Crystal Formation and Kidney Injury Through Histone Lactylation‐Mediated FGFR4 Activation

**DOI:** 10.1002/advs.202413937

**Published:** 2025-02-04

**Authors:** Zehua Ye, Yushi Sun, Songyuan Yang, Lei Li, Bojun Li, Yuqi Xia, Tianhui Yuan, Weimin Yu, Lijia Chen, Xiangjun Zhou, Fan Cheng

**Affiliations:** ^1^ Department of Urology Renmin hospital of Wuhan university Wuhan 430060 China

**Keywords:** CaOx crystal, epigenetics, histone lactylation, kidney injury, Lgals3

## Abstract

The incidence of kidney stones is increasing worldwide. However, the underlying mechanism of the process of kidney stone formation and the kidney damage caused are not well understood. Here, it is observed that Lgals3, a β‐galactoside‐binding protein, is significantly increased in tissues with calcium oxalate (CaOx) stones, and in both in vivo and in vitro models. Lgals3 expression is positively correlated with the deposition of CaOx crystals. Knockout of Lgals3 markedly reduces the deposition of CaOx crystal and renal fibrosis in vivo. Furthermore, Lgals3 deficiency decrease the glycolytic rate and lactate production during the process of CaOx deposition and inhibited histone lactylation of H3K18la. Mechanistic studies shows that Lgals3 directly interacted with the key glycolysis protein pyruvate kinase M2 (PKM2) and promoted its expression by modulating E3 ligase Trim21, preventing the ubiquitination of PKM2. Furthermore, H3K18 lactylation promoted CaOx crystal deposition and kidney injury in vivo and in vitro. Lgals3 deficiency inhibites the transcription, activation, and expression of FGFR4 through inhibition of H3K18la. These findings suggest that Lgals3 may play a key role in CaOx stone formation and kidney injury by interacting with PKM2 and promoting both H3K18la‐mediated gene transcription and activation.

## Graphical abstract

This study indicates that Lgals3 plays a potential novel therapeutic target for the prevention of Calcium oxalate (CaOx) stone formation and subsequent renal fibrosis. Elevated Lgals3 interacted with PKM2 and promote the expression of FGFR4 via histone H3K18 lactylation, thereby facilitating CaOx crystal deposition and the development of renal fibrosis.

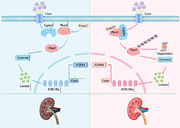

## 1 Introduction

Kidney stones are among the most prevalent urinary system diseases, with their incidence rising globally.^[^ ^1^ ^]^ While most patients opt for stone removal through surgery, studies have shown that this method has up to a 35% recurrence rate.^[^ ^2^ ^]^ Calcium oxalate (CaOx) stones account for approximately 75% of patients with kidney stones^[^ ^3^ ^]^ CaOx stone formation is primarily caused by an imbalance of crystallization, which involves a complex series of processes that begins with nucleation.^[^ ^4^ ^]^ The deposition of crystals in the kidney induces the production of reactive oxygen species and the aggregation of inflammatory factors, leading to the damage of renal tubular epithelial cells.^[^ ^5^ ^]^ The damaged cells exacerbate the aggregation of crystals, ultimately resulting in the formation of kidney stones, decline of renal function, and eventually renal fibrosis.^[^ ^6^ ^]^

Galactose lectin 3 (Lgals3) is a β‐galactoside‐binding lectin that binds to β‐galactosides via its carbohydrate‐recognition structural domain.^[^ ^7^ ^]^ Epidemiological research indicates that higher levels of plasma or serum Lgals3 are linked to reduced renal function and a heightened risk of developing chronic kidney disease (CKD).^[^ ^8^ ^]^ In the presence of tissue damage, evidence suggests that Lgals3 can modulate a variety of glycosylated matrix proteins, such as laminin, fibronectin, and integrins, to promote cell adhesion and proliferation, leading to tissue fibrosis.^[^ ^9^ ^]^ In addition, a study found that Lgalse3 in a unilateral ureteral obstruction model promoted the M2 macrophage polarization that led to renal fibrosis.^[^ ^10^ ^]^ However, the underlying mechanism of Lgals3 in CaOx kidney formation is unclear.

The kidneys are rich in mitochondria that produce large quantities of ATP to provide energy for cellular processes, including renal transport and reabsorption.^[^ ^11^, ^12^ ^]^ Research has demonstrated the significance of metabolic reprogramming in acute kidney injury (AKI) and chronic kidney disease (CKD).^[^ ^13^ ^]^ Under renal ischemic and hypoxic conditions, fatty acid oxidation is inhibited, resulting in a reduction of ATP biosynthesis. In addition, the glycolytic pathway displays progressive upregulation in response to sustained renal injury.^[^ ^14^ ^]^ This metabolic shift exhibits a double‐edged role. Primarily, it provides the kidneys with essential ATP to maintain renal function. However, it can lead to renal lactic acid accumulation and lipid deposition, contributing to the progression of renal fibrosis.^[^ ^15^ ^]^ Traditionally, lactic acid was considered a metabolic waste product of glycolysis during hypoxia. However, Zhao et al found that lactic acid plays an important role in posttranslational modifications by lactylating histone and non‐histone proteins.^[^ ^16^ ^]^ Histone lactylation controls downstream genes' transcription by modulating open regions of chromatin, similar to histone methylation and acetylation.^[^ ^17^, ^18^, ^19^ ^]^ Previous studies that evaluated sepsis‐associated AKI models found that histone H3K18 lactylation was elevated and promoted the transcription of RhoA, leading to inflammation, cell apoptosis, and kidney dysfunction.^[^ ^20^ ^]^ However, the role of lactylation in CaOx crystal formation and renal fibrosis remains to be explored.

In the present study, RNA‐seq, immunoprecipitation mass spectrum, and CUT&Tag were utilized. The results revealed that Lgals3‐mediated lactate generation promoted lactylation at H3K18, which contributed to FGFR4 expression, and promoted CaOx kidney stone formation and renal fibrosis. These findings suggest that Lgals3 and metabolic‐related lactylation may be potential therapeutic targets for patients with kidney stones and offer novel insights into the mechanism to prevent the development of stone formation.

## 2 Results

### 2.1 Lgals3 is Markedly Upregulated in Both Mouse and Human Kidney Stone Specimens

Kidney tissues from CaOx kidney stone patients and control kidney tissues from renal carcinoma patients were collected for RNA‐seq (Figure S1A, Supporting Information). In total, 2728 differentially expressed genes (DEGs) were identified, with 1768 genes showing upregulation and 960 genes showing downregulation (Figure S1B, Supporting Information). The Kyoto Encyclopedia of Genes and Genomes (KEGG) and Gene Ontology (GO) enrichment analysis based on the DEGs found that the upregulated genes were closely associated with extracellular matrix (ECM) and ECM‐receptor interaction (Figure S1C,D, Supporting Information). Then, we established an CaOx stone mouse model (Figure S1E, Supporting Information). HE, Von‐kossa staining and Western blot analysis successfully validated the establishment of the model (Figure S1F—H, Supporting Information). We collected the kidney tissues and performed RNA‐seq and four‐dimensional independent data acquisition (4D‐DIA) proteomic analyses (Figure S1I, Supporting Information).

**Figure 1:**
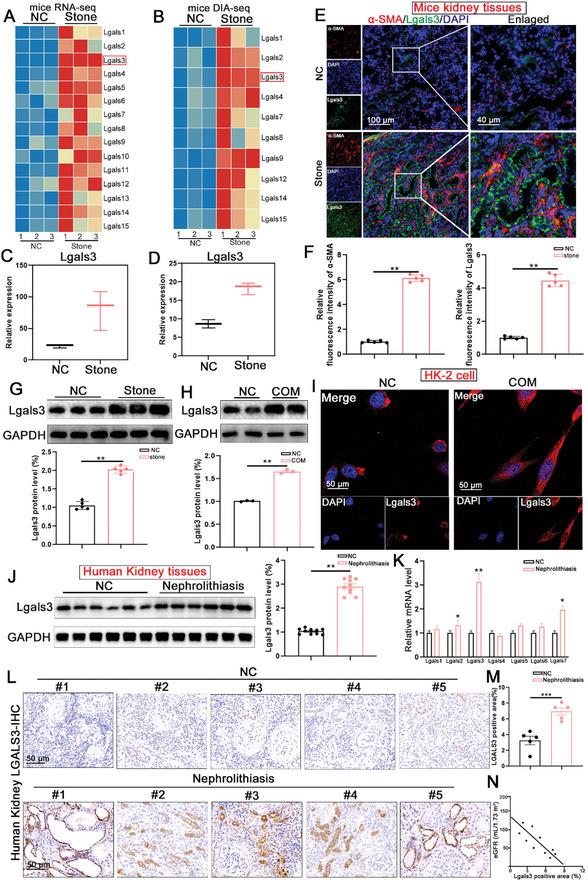
Expression and distribution of Lgals3 in both mouse and human CaOx stone specimens. A) heatmap for differentially expressed Lgals family member in the kidney tissues from NC mice and CaOx stone mice based on the RNA‐seq. B) heatmap for differentially expressed Lgals family member in the kidney tissues from NC mice and CaOx stone mice based on the DIA proteomic analysis. C) The expression of Lgals3 based on the result of RNA‐seq. D) The expression of Lgals3 based on the DIA proteomic analysis. E‐F) The immunofluorescence images and quantification of Lgals3 and α‐SMA levels in the kidney tissues from NC mice and CaOx stone mice (scale bar = 100 µm in first colcumn and 40 µm in second colcums, n = 5 mice per group). G) Immunoblots of the protein expression levels of Lgals3 in kidney tissues from NC mice and CaOx stone mice (n = 5 mice per group). H) Immunoblots of the protein expression levels of Lgals3 in HK‐2 cells treated with COM for 48h. I) Immunofluorescence images showing the Lgals3 level in HK‐2 cell with COM treated for 48h. J) Immunoblots of the protein expression levels of Lgals3 in kidney tissues from CaOx kidney stone patients (n = 10 per group). K) mRNA levels of Lgals1‐Lgals7 in kidney tissues from CaOx kidney stone patients (n = 10 per group). L,M) Immunohistochemical staining and quantification of Lgals3 in kidney tissues from CaOx kidney stone patients. (scale bar = 50 µm, n = 10 per group). N) The correlation between Lgals3 expression and kidney function (eGFR) in the clinical cohort. *P<0.01; **P<0.05.

To explore the role of Lgals family in the process of kidney injury caused by CaOx crystal, individual members of the Lgals family were characterized and quantified based on the RNA‐seq and 4D‐DIA proteomic analyses. The results found that Lgals3 exhibited the most pronounced upregulation in the kidney from CaOx stone mice (**Figure** **1**A–D). Immunofluorescence staining and Western‐blot analysis further confirmed increased Lgals3 levels in vivo and in vitro (Figure 1E–I). Meanwhile, we co‐stained the Lgals3 with proximal tubules and found that Lgals3 was mainly increased in proximal tubules (Figure S2A, Supporting Information).

Then, a single‐nucleus atlas of the human nephrolithiasis database (GSE231568) was used to test the expression of Lgals3. As is shown in the Figure S2B (Supporting Information), the cell was divided into 9 different types such as proximal tubule cell, mastcell and endothelial. Then, we evaluated the Lgals3 expression and the results showed that Lgals3 was significantly increased in the kidney of nephrolithiasis patients (Figure S2C—E, Supporting Information). In addition, the upregulation of Lgals3 was further confirmed in the kidneys of nephrolithiasis patients by western blot and qPCR (Figure 1J–K).To assess the clinical relevance of Lgals3 in CaOx stone, 10 kidney tissues from patients with CaOx kidney stone patients and 10 kidney tissues from radical nephrectomy for renal cell carcinoma were analyzed, showing a significant increase in Lgals3 expression in patients with kidney stone, which correlated closely with kidney function (Figure 1L–N).

### 2.2 Lgals3 Deficiency Inhibits Kidney Injury and Renal Fibrosis Caused by CaOx Crystal

**Figure 2:**
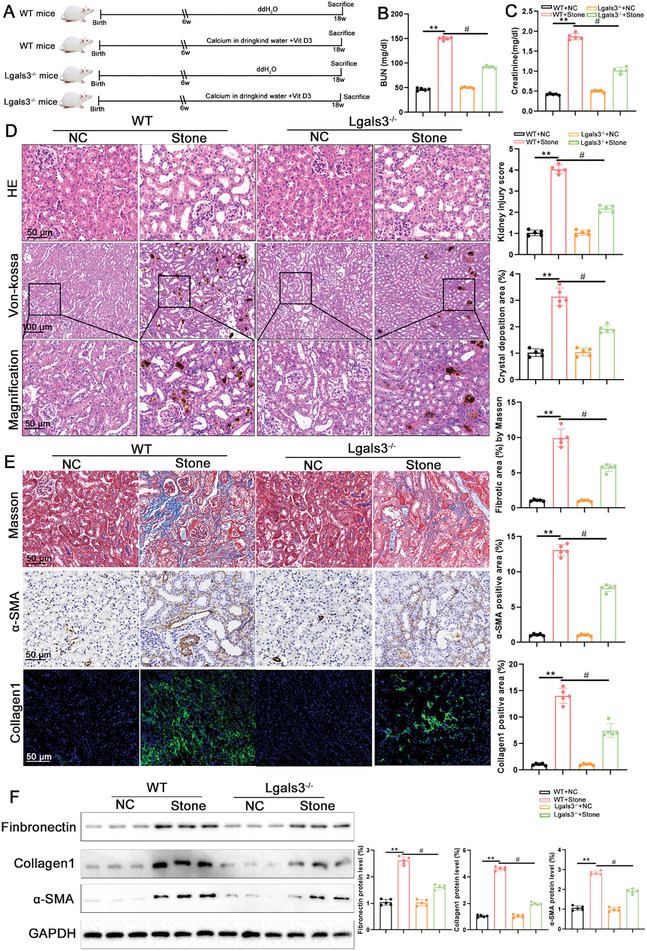
Lgals3 deficiency inhibits kidney injury and renal fibrosis caused by CaOx crystal. A) The schematic of the experimental design. B‐C) The BUN and Scr level in blood from WT mice and Lgals3 knockout (Lgals3^−/−^) mice (n = 5 mice per group). D) Representative images and quantification of HE and Von‐kossa staining in kidney tissues from WT mice and Lgals3 knockout (Lgals3^−/−^) mice (scale bar = 50 µm, n = 5 mice per group). E) Representative images and quantification of Masson staining and the immunohistochemical staining of α‐SMA and Collagen1 in kidney tissues from WT mice and Lgals3 knockout (Lgals3^−/−^) mice (scale bar = 50 µm, n = 5 mice per group). F) Immunoblots of the protein expression levels and quantification of Fibronectin, α‐SMA and Collagen1 in kidney tissues from WT mice and Lgals3 knockout (Lgals3^−/−^) mice (n = 5 mice per group). **P<0.01, compared to the NC group; ^#^ *P*<0.05, compared with WT‐Stone mice.

To explore the role of Lgals3 in kidney injury caused by CaOx crystal, Lgals3 knockout (Lgals3^−/−^) mice were generated (Figure S3A,B, Supporting Information). qPCR, Western blot and immunohistochemistry staining were used to verify knockout efficiency (Figure S3C—E, Supporting Information). Subsequently, a CaOx kidney stone model was established (**Figure** **2**A). CaOx crystal deposition markedly elevated blood urea nitrogen (BUN) and creatinine levels, whereas Lgals3 knockout ameliorated kidney function (Figure 2B,C). Histological assessment of kidney pathology by hematoxylin and eosin (HE) and Von Kossa staining showed that tubular injury and CaOx crystal deposition were significantly increased in the model group, whereas knockout of Lgals3 alleviated kidney injury and CaOx crystal formation (Figure 2D). Meanwhile, renal fibrotic structural alterations were evaluated, and Lgals3 knockout reduced collagen fiber deposition (Figure 2E,F). In addition, Inflammatory responses play a crucial role in the formation of calcium oxalate kidney stones. We found that CaOx crystals leads to an increase in the expression of inflammatory cytokines such as *IL‐1*,*IL‐6* and *TNF‐α* (Figure S3F, Supporting Information). Conversely, the knockout of Lgals3 results in a significant decrease in the expression of these inflammatory factors.

To identify the role of Lgals3 in vitro, a Lgals3‐knockdown HK‐2 cell line was established and western blot analysis confirmed that Lgals3 knockdown was successful (Figure S4A, Supporting Information). The HK‐2 cells were treated with COM for 48h to established the cell model. Immunofluorescence staining found that Lgals3 knockdown alleviated α‐SMA expression in COM‐treated HK‐2 cells (Figure S4C, Supporting Information). Western blot analysis showed a significant decrease in fibrosis‐related protein expression under the stimulation of COM in vitro (Figure S4D, Supporting Information). In addition, the immunofluorescence staining of ROS showed that COM significantly increased the level of ROS, while knockdown of Lgals3 decreased the level of ROS (Figure S4E, Supporting Information). These results suggest that deficiency of Lgals3 inhibits CaOx crystal deposition and renal fibrosis in vivo and in vitro.

### 2.3 Lgals3 Overexpression Promotes Kidney Injury and Renal Fibrosis Induced by CaOx Crystal

**Figure 3:**
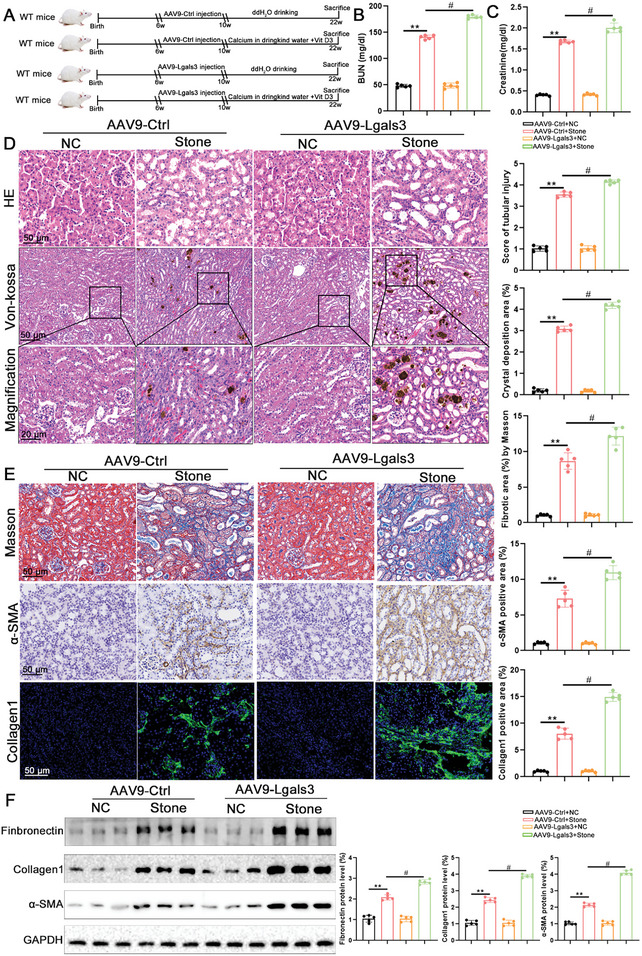
Lgals3 overexpression promotes kidney injury and renal fibrosis induced by CaOx crystal. A) The schematic of the experimental design. B,C) The BUN and Scr level in blood from AAV9‐NC mice and AAV9‐Lgals3 mice (n = 5 mice per group). D) Representative images and quantification of HE and Von‐kossa staining in kidney tissues from AAV9‐NC mice and AAV9‐Lgals3 mice (scale bar = 50 um, n = 5 mice per group). E) Representative images and quantification of Masson staining and the immunohistochemical staining of α‐SMA and Collagen1 in kidney tissues from AAV9‐NC mice and AAV9‐Lgals3 mice (scale bar = 50 um, n = 5 mice per group). F) Immunoblots of the protein expression levels and quantification of Fibronectin, α‐SMA and Collagen1 in kidney tissues from AAV9‐NC mice and AAV9‐Lgals3 mice (n = 5 mice per group). **P<0.01, compared to the NC group; ^#^ *P*<0.05, compared with AAV9‐Ctrl‐Stone mice.

To investigate whether Lgals3 promoted kidney injury and renal fibrosis caused by CaOx crystal, a kidney‐specific overexpression system that overexpressed Lgals3 was utilized in mice (Figure S5A, Supporting Information). After four weeks of injection, the effectiveness of Lgals3 overexpression was confirmed by qPCR and Western blot (Figure S5B,C, Supporting Information). Subsequently, a CaOx kidney stone model was established (**Figure** **3**A). The serum BUN and Creatinine results showed that Lgals3 overexpression migrates the kidney injury (Figure 3B,C). HE and Von Kossa staining found that Lgals3 overexpression aggravated kidney injury and CaOx crystal deposition (Figure 3D). In addition, pathology staining and Western blot analysis found that overexpression of Lgals3 significantly increased renal fibrosis and inflammation response caused by CaOx crystal deposition (Figure 3E,F; Figure S5D, Supporting Information).

An adenoviral vector encoding the Lgals3 gene was utilized in vitro to investigate the effects of Lgals3 overexpression. The overexpression efficiency was confirmed by Western blot (Figure S4B, Supporting Information). Western blot analysis and immunofluorescence staining of ROS found that overexpression of Lgals3 significantly increased the expression of fibrosis‐related protein and ROS expression (Figure S4F,G, Supporting Information). Taken together, these results indicate that Lgals3 overexpression can promote CaOx crystal deposition and renal fibrosis.

### 2.4 Lgals3 Regulates Glycolysis During the Process of Kidney Injury Caused by CaOx Crystal

**Figure 4:**
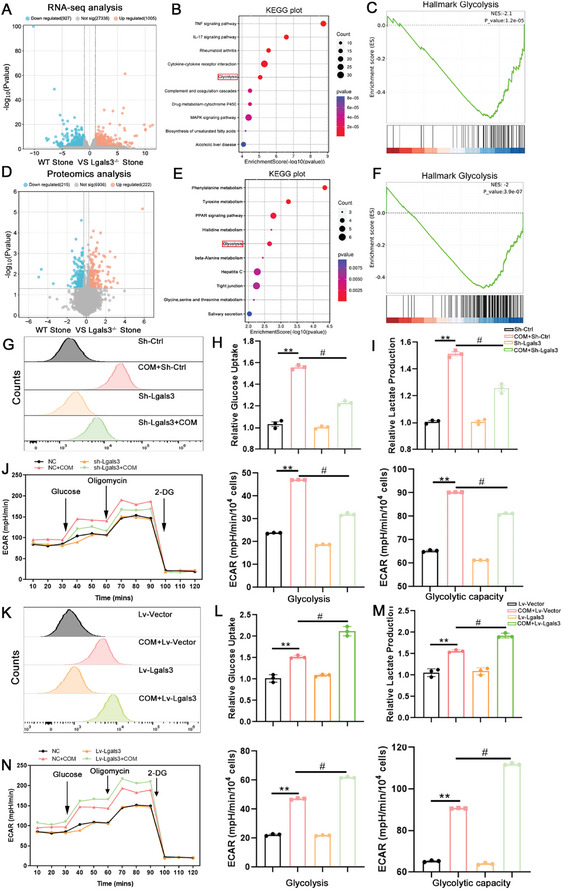
Lgals3 regulates glycolysis during the process of kidney injury caused by CaOx crystal. A) Volcano plot for differentially expressed genes (DEGs) in the kidney tissues from WT mice and Lgals3 knockout (Lgals3^−/−^) mice that with CaOx crystal deposition. (n = 5 mice per group). B) Bubble chart showing the KEGG pathway enrichment analysis of the DEGs. C) Gene Set Enrichment Analysis (GSEA) of WT and Lgals3^−/−^ mice from RNA‐seq data. D) Volcano plot for differentially expressed proteins (DEPs) in the kidney tissues from WT mice and Lgals3 knockout (Lgals3^−/−^) mice (n = 5 mice per group). E) Bubble chart showing the KEGG pathway enrichment analysis of the DEPs. F) Gene Set Enrichment Analysis (GSEA) of WT and Lgals3^−/−^ mice from DIA proteomic data. G) The flow cytometry was used to monitor glucose uptake in Sh‐ctrl HK‐2 and Sh‐Lgals3 HK‐2 cells incubated with 2‐NDBG. H,I) The glucose uptake and lactate production in Sh‐ctrl HK‐2 and Sh‐Lgals3 HK‐2 cells. J) Seahorse metabolic analysis to test the glycolysis in Sh‐ctrl HK‐2 and Sh‐Lgals3 HK‐2 cells. **P<0.01, compared to the Sh‐Ctrl group group; ^#^ *P*<0.05, compared with COM + Sh‐Ctrl group. K) The flow cytometry was used to monitor glucose uptake in Lv‐Vector HK‐2 and Lv‐Lgals3 HK‐2 cells incubated with 2‐NDBG. L,M) The glucose uptake and lactate production in Lv‐Vector HK‐2 and Lv‐Lgals3 HK‐2 cells. N) Seahorse metabolic analysis to test the glycolysis in Lv‐Vector HK‐2 and Lv‐Lgals3 HK‐2 cells. **P<0.01, compared to the Lv‐Vector group; ^#^ *P*<0.05, compared with COM +Lv‐Vector group.

To explore the underlying mechanism of Lgals3 in kidney injury caused by CaOx crystal, RNA‐seq and 4D‐DIA proteomic analysis was performed on Lgals3^−/‐^ and WT mice that with CaOx crystal deposition. RNA‐seq results identified a total of 1932 DEGs, with 1005 exhibiting significant upregulation and 927 exhibiting significant downregulation (**Figure** **4**A). The KEGG analysis showed that inflammation‐related and glycolysis‐related pathways were significantly enriched based on the DEGs, and the gene set enrichment analysis (GSEA) analysis showed that Lgals3 knockout inhibited glycolysis‐related genes at the RNA level (Figure 4B,C). Moreover, 4D‐DIA proteomic results showed a total of 437 DEPs, with an upregulation of 222 and a downregulation of 215 proteins (Figure 4D). The KEGG and GSEA enrichment analysis also showed that glycolysis‐related pathways were significantly enriched, and were subsequently inhibited after Lgals3 knockout (Figure 4E,F). To validate these results, 2‐NDBG glucose uptake detection and lactate production assays were performed. The results indicated that the knockdown of Lgals3 reduced both glucose uptake and lactate production (Figure 4G,I). Conversely, Lgals3 overexpression promoted glucose uptake and lactate production (Figure 4K–M). Meanwhile, Seahorse glycolytic rate analysis found that COM‐treated cells exhibited an increase in basic and compensatory glycolysis, whereas Lgals3 knockdown decreased the basic and compensatory glycolysis (Figure 4J). Conversely, overexpression of Lgals3 promoted glycolytic activity (Figure 4K). In addition, HK‐2 cells exposed to COM showed that knockdown Lgals3 showed no significantly difference in basal respiration oxygen consumption rate, maximal respiration oxygen consummation rate, indicated that Lgals3 knockdown do not affect oxidative phosphorylation (Figure S6, Supporting Information).

### 2.5 Lgals3 Interacts with PKM2 and Regulates PKM2 Level to Promote Lactate Generation

**Figure 5:**
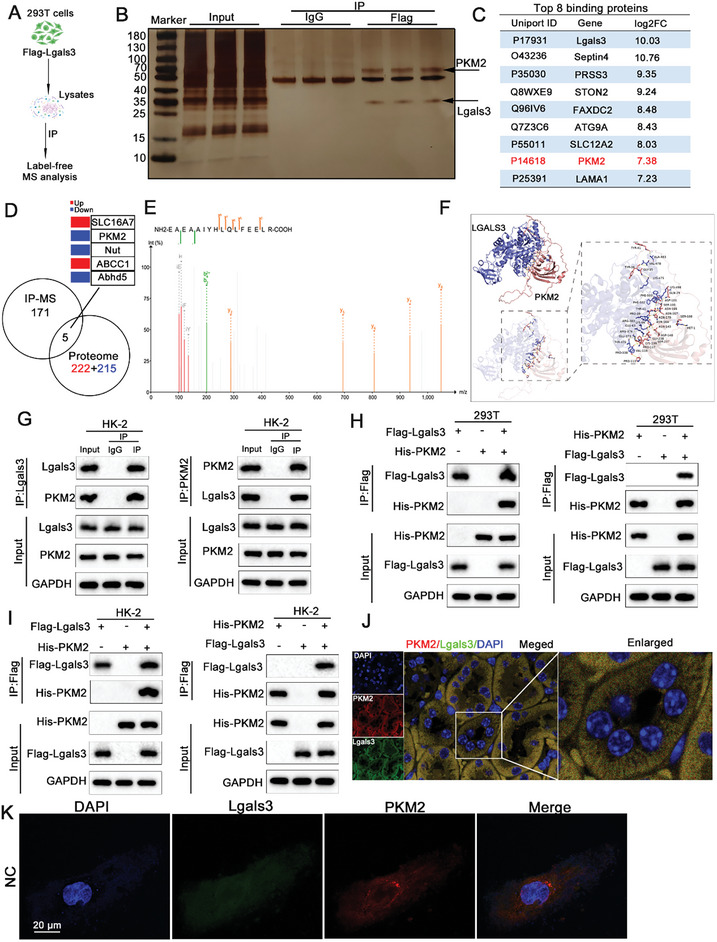
Lgals3 interacts with PKM2 and regulates PKM2 level to promote lactate generation. A) The schedule of IP‐MS. B) Silver staining showing IP products. C) The top8 proteins that binding to Lgals3. D) The overlap protein from the IP‐MS database and DIA protemic analysis. E) The peptide of PKM2 from IP‐MS. F) The molecular docking of Lgals3 and PKM2. G) Cell lysates of HK‐2 cells were immunoprecipitated with Lgals3 or PKM2 antibodies, and immunoblot assays were performed. H) 293T cells were transfected with plasmids encoding Flag‐Lgals3 or His‐PKM2. Cell lysates were immunoprecipitated with Flag and HA antibodies and immunoblot assays were performed. I) HK‐2 cells were transfected with plasmids encoding Flag‐Lgals3 or His‐PKM2. Cell lysates were immunoprecipitated with Flag and HA antibodies and immunoblot assays were performed. J) Representative images of the immunofluorescence staining of Lgals3 (green) and PKM2 (red) in kidney tissues. K) Co‐localization of Lgals3 (green) and PKM2 (red) in the HK‐2 cells.

To determine the direct target of Lgals3 that regulates glycolytic function, whole‐cell lysates were subjected to immunoprecipitation (IP) using Flag antibodies. Protein bands were visualized utilizing silver staining and the products were analyzed using mass spectrometry (IP‐MS; **Figure** **5**A–C). A total of 171 interacting proteins were identified which were cross‐referenced with the DEPs obtained in the 4D‐DIA proteomics sequencing, revealing 5 differentially expressed proteins (Slc16A7, PKM2, Nut, ABCC1, and Abhd5) that bind to Lgals3 (Figure 5D). Among the Lgals3‐interacting proteins, PKM2 was of particular interest as it represented one of the key regulatory enzymes of the glycolytic pathway (Figure 5E). Molecular docking simulations of Lgals3 with PKM2 revealed that Lgals3 could binds to PKM2 (Figure 5F). Co‐IP assays were conducted in HK‐2 cells to further verify Lgals3 and PKM2 binding (Figure 5G). In addition, Co‐IP assays with epitope‐tagged proteins were conducted in HK‐2 and 293T cells. Flag‐Lgals3 and His‐PKM2 were shown to co‐precipitate in 293T and HK‐2 cells (Figure 5H,I). Moreover, Lgals3 and PKM2 exhibited co‐localization in the kidney and HK‐2 cells (Figure 5J,K).

The role of PKM2 in renal fibrosis caused by CaOx crystal was explored further. To investigate the functional role of PKM2, an siRNA‐mediated knockdown of PKM2 was employed, and the results demonstrated that inhibition of PKM2 alleviated renal fibrosis in vitro (Figure S7A,B, Supporting Information). In addition, the western blot analysis confirmed that the expression of PKM2 was decreased after the knockout of Lgals3 in vivo and in vitro (Figure S7C,D, Supporting Information). Then, a rescue assay was performed and found that Lgals3 knockdown decreased the expression of fibrosis‐related proteins. However, the overexpression of PKM2 restored the expression of these proteins in vitro (Figure S7E,F, Supporting Information).

### 2.6 Lgals3 Inhibits PKM2 Ubiquitination and Degradation by Interact with Trim21

**Figure 6:**
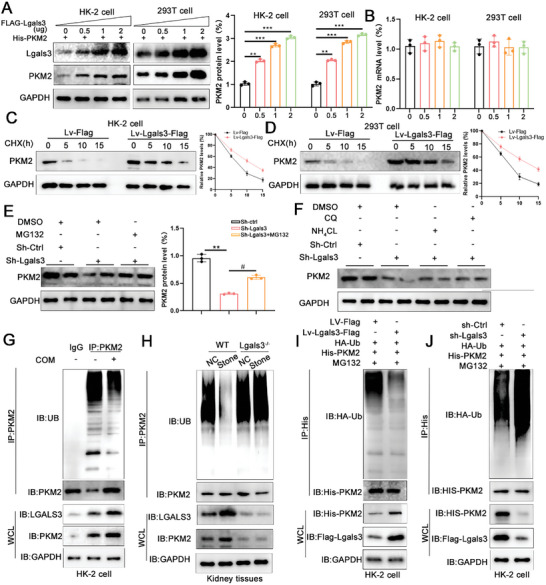
Lgals3 inhibits PKM2 Ubiquitination and degradation. A) Immunoblots of the protein expression levels of PKM2 in HK‐2 and 293T cells transfected with the indicated doses of plasmids encoding Flag‐Lgals3. **P<0.01, ***P<0.005, compared to the NC group. B) The mRNA level of PKM2 in HK‐2 and 293T cells transfected with the indicated doses of plasmids encoding Flag‐Lgals3. C) Cycloheximide (CHX) was used to block protein synthesis. Immunoblots of the protein expression levels and quantification of PKM2 in HK‐2 cells. D) Cycloheximide (CHX) was used to block protein synthesis. Immunoblots of the protein expression levels and quantification of PKM2 in 293T cells. E) Immunoblots of the protein expression levels and quantification of PKM2 in MG132 treated HK‐2 cells (n = 3 per group). F) Immunoblots of the protein expression levels and quantification of PKM2 in CQ and NH_4_CL treated HK‐2 cells (n = 3 per group). **P<0.01, compared to the Sh‐Ctrl group; ^#^ *P*<0.05, compared with Sh‐Lgals3 group. G) Detection of endogenous ubiquitination levels of PKM2 in COM treated HK‐2 cells. H) Detection of endogenous ubiquitination levels of PKM2 in kidney tissues of CaOx mice. I,J) Cells were pretreated with MG132 (5 um) for 8h. Detection of the ubiquitinated levels of PKM2 in Lgals3‐overexpression or Lgals3‐knockdown HK‐2 cells.

Next, we explored how Lgals3 regulates PKM2. Using western blot and qPCR assays, we found that Lgals3 treatment increases the PKM2 protein level in HK‐2 and 293T cells, but had no impact on mRNA level (**Figure** **6**A,B). Therefore, we hypothesized that Lgals3 may regulate PKM2 protein levels via posttranslational modifications. To confirm this hypothesis, control HK‐2 cells and overexpressing Lgals3 HK‐2 cells were treated with cycloheximide, an inhibitor of protein synthesis. The results showed that Lgals3 overexpression prolonged the half‐life of PKM2 (Figure 6C,D). Subsequently, MG132, a proteasome inhibitor, was administered, which restored PKM2 protein levels in Lgals3 knockdown HK‐2 cells (Figure 6E). However, the lysosomal inhibitor chiloroquine (CQ) and NH_4_CL do not restored PKM2 protein levels, indicating that Lgals3 regulates the expression of PKM2 through the ubiquitination pathway (Figure 6F). Further examination of ubiquitination levels was conducted in both in vivo and in vitro models. The findings demonstrated a significant decrease in PKM2 ubiquitination levels in COM‐treated HK‐2 cells compared to the controls (Figure 6G). Similarly, in the CaOx stones mice model, the ubiquitination level of PKM2 was significantly decreased, which was restored by Lgals3 knockout (Figure 6H). In addition, overexpression or knockdown Lgals3 in HK‐2 cells decreased or increased PKM2 ubiquitination, respectively (Figure 6I,J). Hence, the above results indicated that Lgals3 regulates the expression of PKM2 through the ubiquitination pathway.

**Figure 7:**
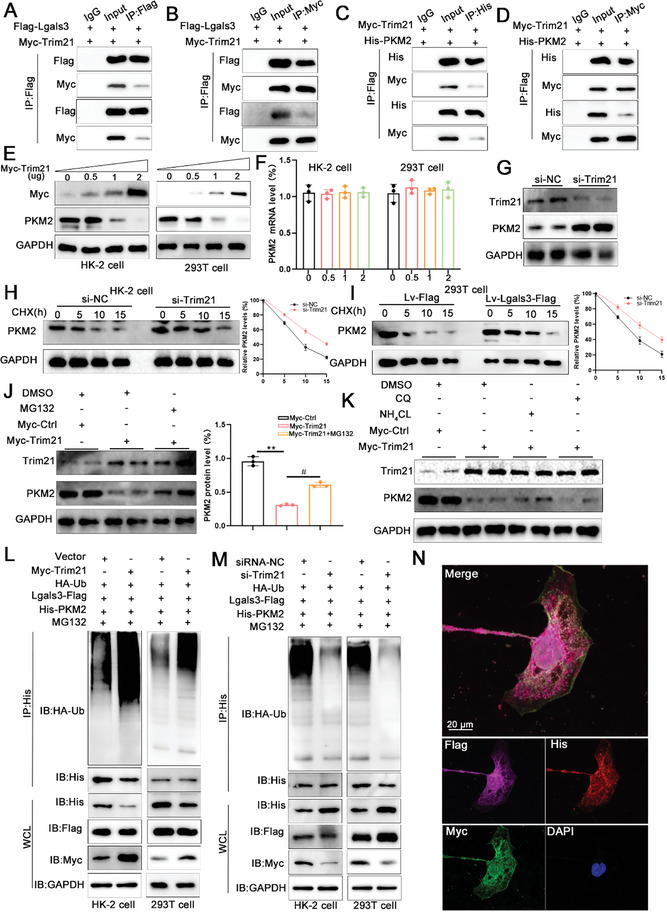
Lgals3 inhibits PKM2 Ubiquitination and degradation by interact with Trim21. A‐D) Cell lysates of HK‐2 cells were immunoprecipitated with Flag, Myc and His antibodies, and immunoblot assays were performed. E) Immunoblots of the protein expression levels of PKM2 in HK‐2 and 293T cells transfected with the indicated doses of plasmids encoding Myc‐Trim21. F) The mRNA level of PKM2 in HK‐2 and 293T cells transfected with the indicated doses of plasmids encoding Myc‐Trim21. G) Immunoblots of the protein expression levels and quantification of PKM2 in Trim21 knockdown HK‐2 cells (n = 3 per group). H) Cycloheximide (CHX) was used to block protein synthesis. Immunoblots of the protein expression levels and quantification of PKM2 in HK‐2 cells. I) Cycloheximide (CHX) was used to block protein synthesis. Immunoblots of the protein expression levels and quantification of PKM2 in 293T cells. J) Immunoblots of the protein expression levels and quantification of PKM2 in MG132 treated HK‐2 cells (n = 3 per group). **P<0.01, compared to the Myc‐Ctrl group; ^#^ *P*<0.05, compared with Myc‐Trim21 group. K) Immunoblots of the protein expression levels and quantification of PKM2 in CQ and NH_4_CL treated HK‐2 cells (n = 3 per group). L‐M) HK‐2 and 293T cells were pretreated with MG132 (5 um) for 8h. Detection of the ubiquitinated levels of PKM2 in Trim21‐overexpression or Trim21‐knockdown cells. N) Co‐localization of Lgals3, PKM2 and Trim21 in HK‐2 cells.

Next, IP‐MS data collected in this study was subsequently searched to identify potential E3 ligases that interacted with Lgals3 and modulated PKM2 stability. Trim21 was identified as the sole E3 ligase that interacted with Lgals3 (Figure 5A,B). Co‐IP assays verified that Trim21 directly interacted with both Lgals3 and PKM2 (**Figure** **7**A–D). Subcellular co‐localization of these proteins in HK‐2 cells also confirmed their relationship (Figure 7N). We found that overexpression of Trim21 decreased the protein level of PKM2 in a dose‐dependent manner, while had no impact on its mRNA level (Figure 7E,F). In contrast, knockdown of Trim21 significantly increased the protein level of PKM2, but had no effect on its mRNA level (Figure 7G). In addition, control HK‐2 cells and knockdown Trim21 HK‐2 cells were treated with cycloheximide and the results showed that Lgals3 overexpression prolonged the half‐life of PKM2 (Figure 7H,I). Furthermore, the decrease of PKM2 protein level induced by Trim21 overexpression could be reversed by MG132, but CQ and NH_4_CL did not have the same effect (Figure 7J,K).

Then, we assessed the effect of Trim21 on the ubiquitination level of PKM2 and the results showed that overexpression of Trim21 enhanced PKM2 ubiquitination, whereas Trim21 knockdown inhibited this process in HK‐2 and 293T cells (Figure 7L,M). These results indicate that Lgals3 inhibits PKM2 Ubiquitination and degradation by interact with Trim21.

### 2.7 Histone Lactylation is Enhanced during CaOx Crystal Formation

**Figure 8:**
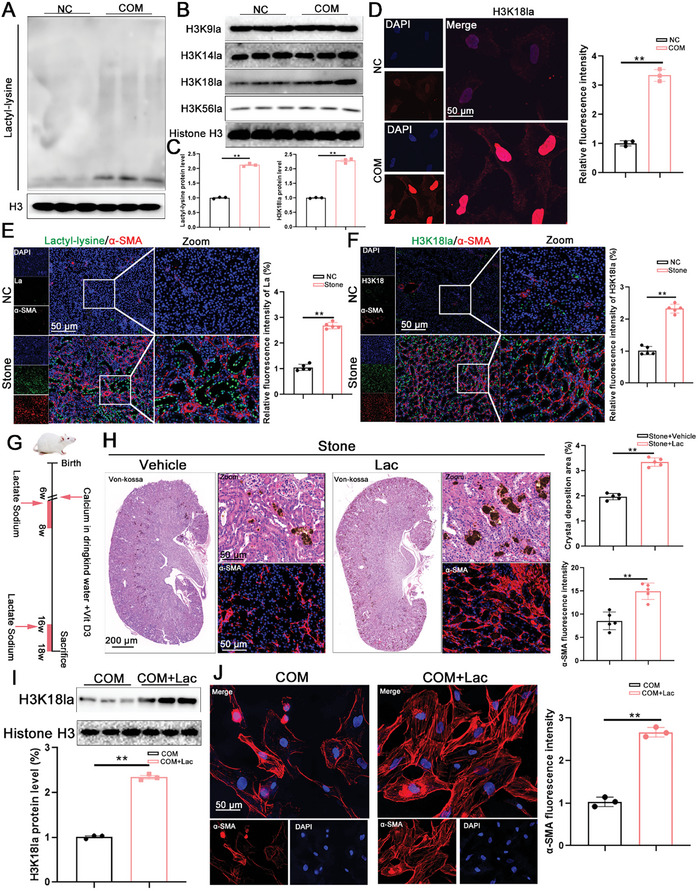
Histone lactylation promotes CaOx crystal formation and renal fibrosis. A) Immunoblots of the protein expression levels and quantification of Lacty‐lysine in HK‐2 cells with COM treated for 48h (n = 3 per group). B,C) Immunoblots of the protein expression levels and quantification of H3K9la, H3K14la, H3K18la and H3K56la in HK‐2 cells with COM treated for 48h (n = 3 per group). D) Immunofluorescence images showing the H3K18la level in HK‐2 cell with COM treated for 48h (n = 3 per group). E) Representative images and quantification of the immunofluorescence staining of Lacty‐lysine and α‐SMA in kidney tissues from NC mice and CaOx mice (scale bar = 50 um, n = 5 mice per group). F) Representative images and quantification of the immunofluorescence staining of H3K18la and α‐SMA in kidney tissues from NC mice and CaOx mice (scale bar = 50 um, n = 5 mice per group). **P<0.01, compared to the NC group. G) The schematic of the experimental design. H) Representative images and quantification of the von‐kossa staining and the immunofluorescence staining of α‐SMA in kidney tissues from mice treated with Lac (scale bar = 50 um, n = 5 mice per group). **P<0.01, compared to the Stone group. I) Immunoblots of the protein expression levels and quantification of H3K18la in HK‐2 cells with Lac treated for 48h (n = 3 per group). J) Immunofluorescence images showing the α‐SMA level in HK‐2 cell with Lac treated for 48h (n = 3 per group). **P<0.01, compared to the COM group.

Lactylation is a recently discovered epigenetic modification that regulates gene expression. In the current study, global lactylation levels were determined in HK‐2 cells under the stimulation of COM. The results showed that global lactylation levels were elevated, and the predominant band observed was 17 kDa, which corresponded to histone H3 (**Figure** **8**A). Next, various lactylation sites on histone H3 and histone H4 were examined, and the level of H3K18 lactylation (H3K18la) increased significantly after COM treatment (Figure 8B,C; Figure S8A,B, Supporting Information). Immunofluorescence analysis also indicated that COM‐stimulated HK‐2 cells exhibited increased levels of H3K18la compared to the controls (Figure 8D). Immunofluorescence was utilized to examine global lactylation and H3K18la levels in CaOx stone mouse model kidney tissues. The analysis confirmed that both global lactylation and H3K18la levels were significantly increased in vivo (Figure 8E,F).

### 2.8 Histone Lactylation Promotes CaOx Crystal Formation and Renal Fibrosis

**Figure 9:**
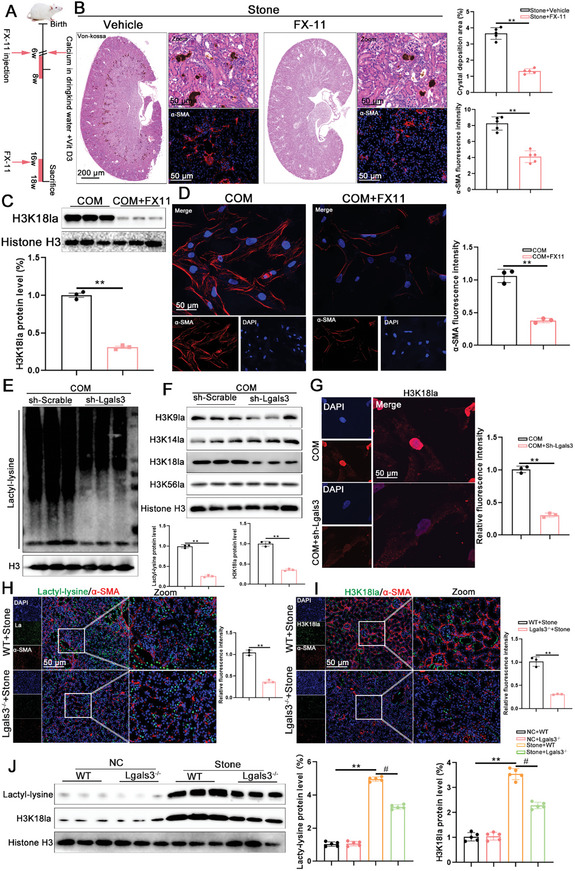
Inhibition of Lgals3 reduces H3K18la during the formation of CaOx crystal. A) The schematic of the experimental design. B) Representative images and quantification of the von‐kossa staining and the immunofluorescence staining of α‐SMA in kidney tissues from mice treated with FX‐11 (scale bar = 50 um, n = 5 mice per group). **P<0.01, compared to the Stone group. C) Immunoblots of the protein expression levels and quantification of H3K18la in HK‐2 cells with FX‐11 treated for 48h (n = 3 per group). D) Immunofluorescence images showing the α‐SMA level in HK‐2 cell with FX‐11 treated for 48h (n = 3 per group). E) Immunoblots of the protein expression levels and quantification of Lacty‐lysine in Lgals3 knockdown HK‐2 cells with COM treated for 48h (n = 3 per group). F) Immunoblots of the protein expression levels and quantification of H3K9la, H3K14la, H3K18la and H3K56la in Lgals3 knockdown HK‐2 cells with COM treated for 48h (n = 3 per group). G) Immunofluorescence images showing the H3K18la level in Lgals3 knockdown HK‐2 cell with COM treated for 48h (n = 3 per group). **P<0.01, compared to the COM group. H) Representative images and quantification of the immunofluorescence staining of Lacty‐lysine and α‐SMA in kidney tissues from WT mice and Lgals3 knockout mice (scale bar = 50 um, n = 5 mice per group). I) Representative images and quantification of the immunofluorescence staining of H3K18la and α‐SMA in kidney tissues from WT mice and Lgals3 knockout mice (scale bar = 50 um, n = 5 mice per group). **P<0.01, compared to the WT+Stone group. J) Immunoblots of the protein expression levels and quantification of Lacty‐lysine and H3K18la in kidney tissues from WT mice and Lgals3 knockout (Lgals3^−/−^) mice (n = 5 mice per group). **P<0.01, compared to the NC group; ^#^ *P*<0.05, compared with WT‐Stone mice.

To assess the effects of histone lactylation on CaOx crystal formation and renal fibrosis, mice were intraperitoneally injected with sodium lactate to induce histone lactylation (Figure 8G). As shown in Figure 8H, lactate supplementation increased CaOx crystal deposition and renal fibrosis, evidenced by Von Kossa and immunofluorescence staining. In vitro, the supplementation of lactate contributed to increased H3K18la levels and α‐SMA expression under COM treatment (Figure 8I,J). Additionally, mice were injected intraperitoneally with FX‐11, a specific inhibitor of LDHA, to suppress histone lactylation (**Figure** **9**A). The pathological staining showed that inhibition of histone lactylation alleviated CaOx crystal deposition and renal fibrosis (Figure 9B). Consistent with the results of the mouse model, the addition of FX‐11 decreased the H3K18la levels and α‐SMA expression after COM treatment (Figure 9C,D).

### 2.9 Inhibition of Lgals3 Reduces H3K18la During CaOx Crystal Formation

Furthermore, this study investigated whether Lgals3 deficiency reduced H3K18la during CaOx deposition. Western blot and immunofluorescence staining showed that Lgals3 knockdown in vitro decreased the levels of global lactylation and H3K18la levels (Figure 9E–G). Similarly, Lgals3^−/−^ mice exhibited significant decreases in global lactylation and H3K18la levels compared with the WT mice through Western blot and immunofluorescence staining (Figure 9H–J). Meanwhile, we also tested the level of acetylation of histone H3 and histone H4 and the results showed that Lgals3 deficiency have no impact on the expression of acetylation of histone H3 and histone H4 (Figure S9, Supporting Information)

### 2.10 Lgals3‐Mediated H3K18la Targets Multiple Fibrosis‐Related Genes

**Figure 10:**
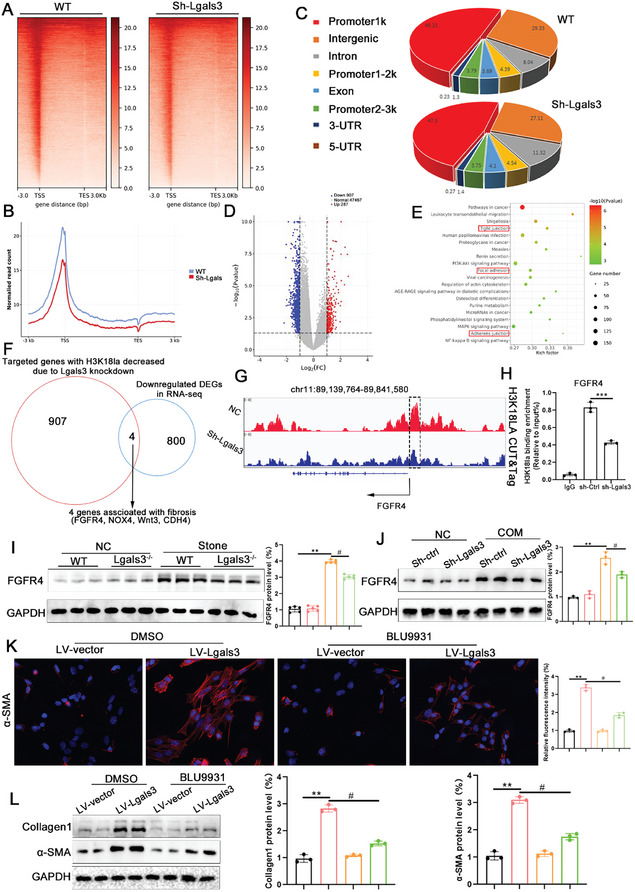
Lgals3‐mediated H3K18la targets multiple fibrosis‐related genes. A,B) The binding density of H3K18la in the transcriptional start site was visualized. C) Genome‐wide distribution of differentiated H4K12la‐binding peaks in Sh‐Ctrl HK‐2 cell and Sh‐Lgals3 HK‐2 cells. D) Volcano plot for different genetic loci bound by H3K18la in CUT‐tag. E) Bubble chart showing the KEGG pathway enrichment analysis of the H3K18la binding DEGs. F) Venn diagram showed genes downregulated in WT and Lgals3 knockout mice and the downregulated target genes bound by H3K18la. G) Integrative Genomics Viewer analysis representing H3K18la peaks at the FGFR4 locus. H) Chip‐qPCR assays of H3K18la in the FGFR4 in HK‐2 cells. I) Immunoblots of the protein expression levels and quantification of FGFR4 in kidney tissues from WT mice and Lgals3 knockout (Lgals3^−/−^) mice (n = 5 mice per group). **P<0.01, compared to the NC group; ^#^ *P*<0.05, compared with WT‐Stone mice. J) Immunoblots of the protein expression levels and quantification of FGFR4 in Lgals3 knockdown HK‐2 cells with COM treated for 48h (n = 3 per group). **P<0.01, compared to the Sh‐Ctrl group; ^#^ *P*<0.05, compared with COM+Sh‐Ctrl group. K) Immunofluorescence images showing the α‐SMA level in Lgals3 overexpression HK‐2 cells with BLU9931 treated for 48h (n = 3 per group). L) Immunoblots of the protein expression levels and quantification of Collagen1 and α‐SMA in Lgals3 overexpression HK‐2 cells with BLU9931 treated for 48h (n = 3 per group). **P<0.01, compared to the Lv‐Vector group; ^#^ *P*<0.05, compared with Lv‐Lgals3 group.

Previous studies have shown that histone lactylation may mediate gene expression. This study employed cleavage under targets and tagmentation (CUT&Tag) targeting H3K18la to characterize genome‐wide changes of histone modifications. Analysis of the CUT&Tag signal enrichment data revealed a distinct heatmap pattern demonstrating that Lgals3 knockdown significantly decreased the H3K18la enrichment signal around the transcription start sites (**Figure** **10**A,B). Meanwhile, global mapping analysis showed that the H3K18la sites were primarily enriched at the promoter regions (Figure 10C). A volcano plot analysis identified a total of 48661 genetic loci bound by H3K18la, of which 907 were downregulated and 287 were upregulated in the Lgals3 knockdown cells compared with the WT HK‐2 cells (Figure 10D). The GO enrichment analysis of the target genes associated with the H3K18la‐binding peaks showed significant enrichment in processes related to tight junctions and focal adhesions (Figure 10E).

To investigate the target gene of H3K18la as a result of Lgals3 deficiency, integrative CUT&Tag and RNA‐seq analysis were utilized. Four overlapping genes were identified: FGFR4, NOX4, Wnt3, and CDH4 (Figure 10F), among which, FGFR4 exhibited the strongest association with fibrosis. Integrative Genomics Viewer analysis of the H3K18la CUT&Tag analysis indicated that H3K18la binds to the FGFR4 promoter region (Figure 10G). In addition, Chip‐qPCR analysis confirmed that H3K18la was significantly enriched on the FGFR4 promoter, whereas Lgals3 knockdown decreased this enrichment (Figure 10H). Concomitantly, qRT‐PCR and Western blot analysis found that the mRNA and protein level of FGFR4 was significantly decreased in Lgals3 deficient mice and in Lgals3 knockdown cells (Figure 10I,J).

To determine whether activation of FGFR4 is required for the pro‐fibrotic effects of Lgals3, an FGFR4 inhibitor, BLU9931, was used in vitro after COM treatment. The increase in α‐SMA expression induced by Lgals3 overexpression was significantly ameliorated after the treatment of BLU9931 (Figure 10K). In addition, Western blot analysis corroborated the findings of the immunofluorescence staining (Figure 10L).

### 2.11 Pharmacological Inhibition of Lgals3 Ameliorates Kidney Injury and Renal Fibrosis Caused by CaOx Crystal

**Figure 11:**
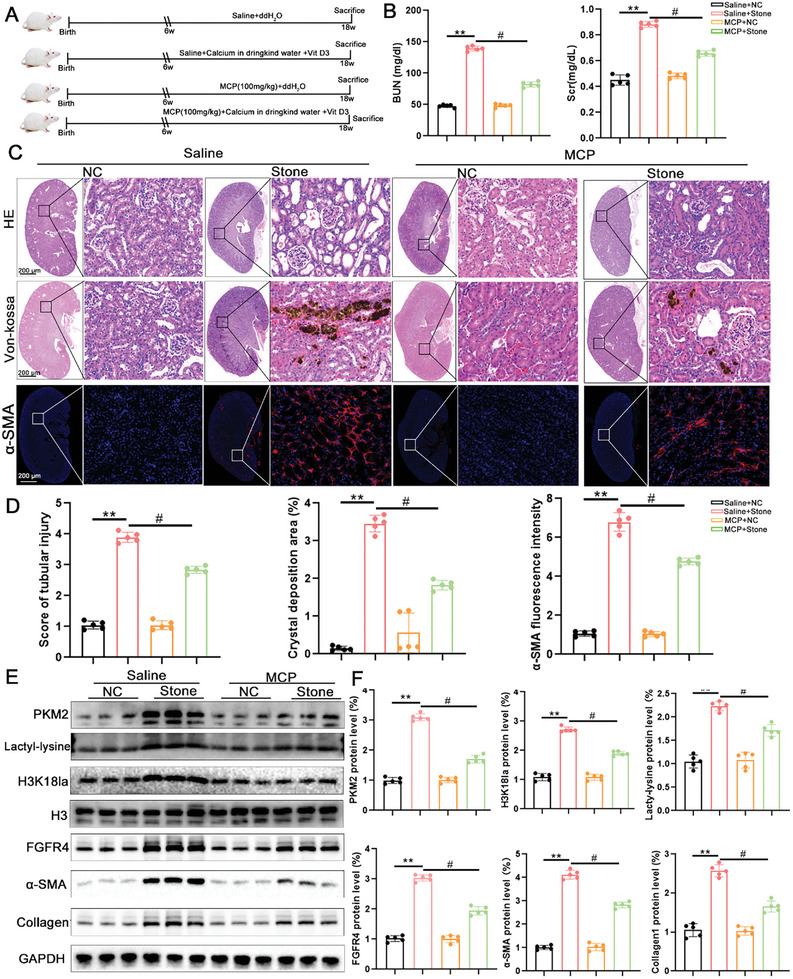
Pharmacological inhibition of Lgals3 ameliorates CaOx crystal formation and renal fibrosis. A) The schematic of the experimental design. B) The BUN and Scr level in blood from Caox stone mice and CaOx stone+MCP mice (n = 5 mice per group). C,D) Representative images and quantification of HE, Von‐kossa and α‐SMA staining in kidney tissues from Caox stone mice and CaOx stone+MCP mice (scale bar = 50 um, n = 5 mice per group). E,F) Immunoblots of the protein expression levels and quantification of PKM2, Lacty‐lysine, H3K18la, FGFR4, α‐SMA and Collagen1in kidney tissues from Caox stone mice and CaOx stone+MCP mice (n = 5 mice per group). **P<0.01, compared to the NC group; ^#^ *P*<0.05, compared with Stone group.

To affirm the therapeutic potential of Lgals3, wild type CaOx stone mice were treated with MCP, an inhibitor of Lgals3 (**Figure** **11**A). The BUN and Scr results showed that MCP administration alleviated kidney injury caused by CaOx crystal (Figure 11B). Histological results showed that MCP significantly alleviated kidney injury, CaOx crystal deposition and renal fibrosis (Figure 11C,D). In addition, western blot analysis affirmed that downregulation of PKM2, FGFR4, α‐SMA and Collagen1 after MCP injection. Furthermore, MCP decreased the global and histone H3K18 lactylation level (Figure 11E,F).

Meanwhile, to verify the clinical potential of targeting Lgals3 for treatment of kidney stones, we established another CaOx crystal mouse model according our previous studies by injecting glycolic acid for consecutively 12 days and inhibited Lgals3 through MCP and GB1107 (Figure S10A, Supporting Information). The results demonstrated that MCP and GB1107 also inhibited the activation of H3K18la in the kidneys of the acute calcium oxalate crystal deposition mice and alleviated both crystal deposition and kidney damage (Figure S10B—E, Supporting Information).

### 2.12 Lgals3 May be Key Factors Involved in CaOx Stone Patients

**Figure 12:**
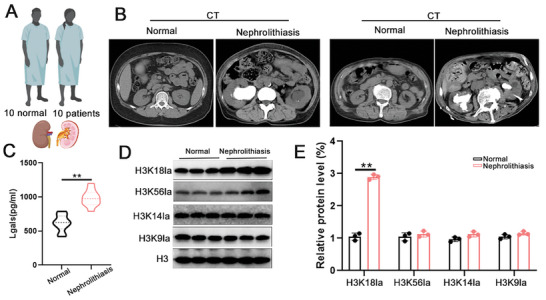
Lgals3 may be key factors involved in CaOx stone patients. A–C) Graphic presentation shows serum Lgals3 levels in a cohort of patients with nephrolithiasis (n = 10) and healthy participants (n = 10). D,E) The H3K18la level in the kidney tissues from CaOx stone patients. **P<0.01 compared to the NC group.

To determine whether serum Lgals3 levels are associated with kidney injury and fibrosis in human patients, we measured serum Lgals3 levels in 10 healthy participants and 10 nephrolithiasis patients (**Figure** **12**A,B). The level of Lgals3 was elevated in nephrolithiasis patients compared with healthy participants (Figure 12C). Furthermore, the HK318la level was also significantly increased in nephrolithiasis patients (Figure 12D,E). These results provide strong evidence that Lgals3 play a key role in the kidney injury caused by CaOx crystal.

## 3 Discussion

**Figure 13:**
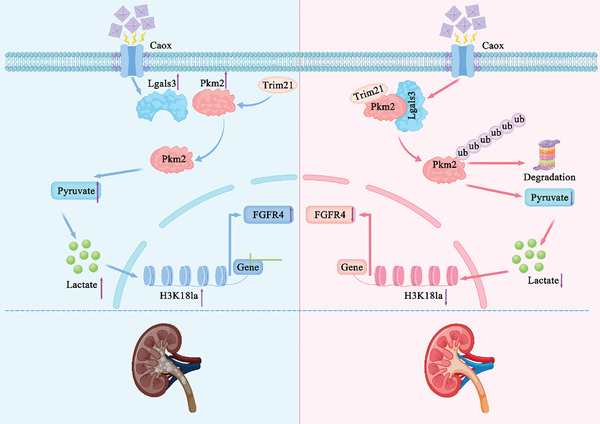
Schematic representation of the mechanism of Lgals3 in CaOx stone formation. Elevated Lgals3 interacted with PKM2 and promoted the expression of FGFR4 via H3K18la, thereby facilitating CaOx crystal deposition and the development of renal fibrosis.

Previous studies have found that Lgals3 plays a crucial role in inflammation, immunity, injury, and fibrosis.^[^ ^21^, ^22^, ^23^ ^]^ The result of the present study revealed that Lgals3 exhibited several novel functions and mechanisms in the formation of kidney stones and the development of renal fibrosis (**Figure** **13**). It was found that Lgals3 was highly expressed in CaOx crystal deposition and stimulated the activation of glycolysis during crystal deposition and renal fibrosis. Knockout or pharmacological inhibition of Lgals3 demonstrated a significant reduction of crystal deposition and renal fibrosis. In addition, IP‐MS analysis identified PKM2, a key molecule in the regulation of glycolytic function, as the direct binding target of Lgals3. Furthermore, this study integrated analyses of CUT&Tag and RNA‐seq and demonstrated that Lgals3‐mediated histone lactylation promoted FGFR4 expression during the formation of CaOx stones and renal fibrosis.^[^ ^24^ ^]^ Lgals3 is considered a disease‐associated biomarker and it is significantly increased in cases of acute myocardial infarction or AKI.^[^ ^25^, ^26^ ^]^ Furthermore, recent investigations have shown that it has significant potential as a therapeutic target for various inflammatory and fibrotic illnesses, including lung or kidney fibrosis.^[^ ^27^ ^]^ In this study, it was found that Lgals3 expression was increased in both mouse and human CaOx crystal kidney tissues. This study utilized Lgals3^−/‐^ mice and demonstrated that Lgals3 deficiency alleviated CaOx crystal deposition and renal fibrosis. The deposition of CaOx crystals and the development of renal fibrosis are complex processes regulated by numerous genes and signaling pathways. RNA‐seq and 4D‐DIA proteomics were performed to detect alterations in mRNA and protein expression in Lgals3‐deficient cells under COM stimulation. The KEGG analysis showed that Lgals3 deficiency primarily enriched metabolic‐related pathways, specifically glycolysis. When cells are exposed to various stimuli, the mitochondrial energy metabolism undergoes alterations, leading to significant activation of glycolysis, which in turn increases the overall energy supply.^[^ ^28^ ^]^ Yang et al have suggested that the knockout of phosphofructokinase 1 (PFKP), an enzyme in glycolysis, in renal tubular epithelial cells inhibited the TGF‐beta pathway, thereby alleviating renal fibrosis.^[^ ^29^ ^]^ In addition, a recent study reported that Lgals3 was overexpressed in several metabolic conditions, such as diabetes, obesity, and atherosclerosis.^[^ ^30^ ^]^ In the current study, it was found that glycolysis was activated after COM stimulation. Further analysis using the Seahorse glycolytic rate confirmed that the knockdown of Lgals3 inhibited the glycolytic flux and reduced glucose uptake and lactate production, suggesting that Lgals3 plays a key role in regulating glycolytic metabolism.

Pyruvate kinase M2 (PKM2) is a crucial enzyme in the glycolysis pathway that controls the rate at which phosphopyruvate is converted into pyruvate and ATP by phosphorylation.^[^ ^31^ ^]^ Xie et al found that PKM2 regulated mitochondrial homeostasis in cisplatin‐induced acute kidney injury by binding to MYH9 to promote dynamin‐related protein 1‐mediated mitochondrial fragmentation, alleviating renal tubular injury and cell death.^[^ ^32^ ^]^ In the present study, it was found that the expression of PKM2 was significantly increased in both in vivo and in vitro models, and the knockdown of PKM2 attenuated CaOx crystal‐induced renal fibrosis. In addition, further analysis showed that Lgals3 promoted PKM2 protein levels by inhibiting ubiquitination and degradation by modulating Trim21. The ubiquitin‐proteasome system (UPS) selectively degrades the majority of cellular proteins through an ATP‐driven process.^[^ ^33^ ^]^ UPS protein degradation has been implicated in various kidney diseases, such as AKI, diabetic nephropathy, and CKD.^[^ ^34^ ^]^ Data from this study suggest that Lgals3 directly interacted with the E3‐ubiquitin ligase Trim21 and prevented PKM2 from undergoing Trim21‐mediated ubiquitination and degradation. These results highlight the importance of the Lgals3‐Trim21‐PKM2 axis in the pathogenesis of renal fibrosis and kidney stone formation.

Lactate was traditionally considered a metabolic waste product produced during glycolysis.^[^ ^35^ ^]^ However, previous studies have found that lactate functions as a precursor which mediate the modification of histone and non‐histone gene expression.^[^ ^36^ ^]^ For instance, previous research suggests that H4K12 lactylation was enriched in the promoter of the NF‐KB signaling pathway, which subsequently activated transcription in kidney tubular cells and aggravated kidney inflammation and fibrosis.^[^ ^37^ ^]^ Observations in the present study found increased global lactylation and histone lactylation levels, particularly H3K18la, during the process of CaOx crystal deposition. Conversely, Lgals3 deficiency inhibited H3K18la activity. A combination of H3K18la‐targeted CUT&Tag and RNA‐seq analyses identified FGFR4 as the key downstream target gene of H3K18la. Additionally, H3K18la was significantly enriched in the promoter region of FGFR4, suggesting that this histone modification may play a role in activating its transcription. FGFR4 is a member of the fibroblast growth factor receptor family and plays a key role in cell survival, proliferation, and migration.^[^ ^38^ ^]^ Previous studies found that FGF23 binds to FGFR4 and increases kidney inflammation. The current study found that FGFR4 was significantly increased and is essential for Lgals3‐mediated CaOx crystal deposition and the development of renal fibrosis.

In conclusion, this study demonstrated the significant role of Lgals3 in the process of CaOx crystal deposition and stone formation. Elevated Lgals3 interacted with PKM2 and promoted the expression of FGFR4 via H3K18la, thereby facilitating CaOx crystal deposition and the development of renal fibrosis (Figure 11). These findings provide a potential novel therapeutic target for the prevention of CaOx stone formation and subsequent renal fibrosis.

## 4 Experimental Section

### Clinical Kidney Tissues

The non‐function human kidney samples were collected from patients diagnosed with kidney stone and control samples from patients diagnosed with renal cell carcinoma in Renmin Hospital of Wuhan University between 2022‐1‐1 and 2024‐1‐1, after obtaining approval from the hospital's Ethics Committee (WDRY2023‐KS033). Normal samples were obtained from healthy kidneys of individuals who underwent tumor nephrectomies without other kidney diseases. The clinical information of the patients was presented in Table S1 (Supporting Information).

### Animal Studies

All experimental procedures received approval from the ethics committee at Renmin Hospital, Wuhan University (WDRM‐2023070). Lgals3 knockout mice (Lgals3^−/−^ mice) were obtained from the Cyagen (Suzou, China). The male C57/BL6 mice were obtained from the Center of Experimental Animals at Wuhan university. All mice were kept in 20–22 with a 12‐hour light‐dark cycle. The control group (C57BL/6J mice) and experimental group (Lgals3^−/−^ mice) mice used in the study were littermates.

Two different types of CaOx crystal mice models, including calcium and 1,25(OH)_2_D_3_‐induced CaOx crystal formation and Glyoxylate acid (Gly)‐induced CaOx crystal formation were used in this study.

For calcium and 1,25(OH)_2_D_3_‐induced CaOx crystal mice model. The mice were given free access to food containing 0.73% calcium and 600IU 1,25(OH)_2_D_3_ and drinking water containing calcium (2g L^−1^) for a period of 3 months to established the CaOx stone model.

For Gly‐induced CaOx crystal mice model. The mice were received an intraperitoneal injection of Gly (120mg k^−1^g d^−1^) for 12 consecutive days to established the CaOx stone model.

### Renal Function

Prior to sacrifice, blood samples were harvested from the orbital sinus of the mice. The collected samples were then analyzed utilizing an automated biochemical analyzer (Beckman, USA) to assess serum Cr, and BUN levels.

### AAV9 Virus Injection

The AAV9 virus were provided by Jiman Biotechnologies. The AAV9 carrying Lgals3 gene with KSP promoter (AAV9‐Lgals3) or the control virus (AAV9‐vector) were injected into 5 different sites of the renal cortex according to the manufacture instruction. One month later, kidney were collected to tested the overexpression efficiency.

### RNA‐Sequencing (RNA‐seq)

This study used the kidney tissues to obtain total RNA for RNA‐Seq analysis. The RNA extraction process involved the use of Trizol reagent, followed by the purification of poly(A) RNA. This purified RNA was then used to form a cDNA library. The sequencing of all samples was performed using the Illumina HiSeq X Ten platform.

### 4D‐DIA Proteomic Sequencing

Kidney tissues were combined with a solution containing 8 M Urea and 100 mM Tris‐Cl, followed by treatment with water bath sonication. Proteins were reduced and alkylated using TCEP and CAA at 37 °C for 1 hour. The urea concentration was then diluted to less than 2 M with 100 mM Tris‐HCl (pH 8.0). Trypsin was added at a 1:50 ratio for overnight digestion at 37 °C. The next day, the digestion process was stopped by adjusting the pH to 6.0 with TFA. After centrifuging at 12 000 g for 15 minutes, the supernatant was processed for peptide purification using a custom‐made SDB‐RPS desalting column. The peptide eluate was vacuum evaporated and stored at ‐20 °C for future use. Samples were analyzed using the timsTOF Pro, a hybrid trapped ion mobility spectrometer (TIMS) quadrupole time‐of‐flight mass spectrometer by Bruker Daltonics. To identify proteins differentially expressed among groups, statistical significance was assessed using an unpaired t‐test.

### Histological Analysis of Kidney Tissues and Immunohistochemical Staining

Kidney tissues were isolated and fixed in 4% paraformaldehyde for 24 hours before being embedded in paraffin. The sections were then stained using Haematoxylin‐eosin (H&E), Von Kossa, and Masson techniques.

For immunohistochemistry, tissue sections were deparaffinized with xylene, ethanol, and water, respectively. Sections were blocked with 10% normal goat serum and incubated with primary antibody against Lgals3 (1:200 dilution) and α‐SMA (1:400 dilution) at 4 °C overnight. After three times washes with TBST, the sections were incubated with DAB for 5 min.

### Cell Culture

Human tubular epithelial cells (HK‐2 cells) were sourced from the Chinese Academy of Sciences Cell Bank. The cells were maintained in DMEM medium supplemented with 10% FBS and 1% antibiotics (penicillin and streptomycin), and cultured in a 37 °C incubator with 5% CO2. To establish the cell model in vitro, HK‐2 cells were exposed to 100 µg ml^−1^ calcium oxalate monohydrate (COM) for 48 hours.

### Cell Transfection

The stably Lgals3 knockdown and Lgals3 overexpression cell lines were constructed by transferring lentivirus into HK‐2 cell according to the manufacturer's instructions (GeneChem, Shanghai).

The expression plasmids encoding Flag‐Lgals3, His‐PKM2, Myc‐Trim21 and HA‐tagged ubiquitin were purchased from Jiman Bio. For transient transfection, plasmids were transfected into HK‐2 or HEK 293T cells with Lipofectamine™ 2000 transfection reagent as required for the experiment.

The PKM2 siRNA (si‐PKM2) and negative control (si‐NC) were acquired from Jiman Bio. The HK‐2 cells were transfected with si‐PKM2 and si‐NC using Lipofectamine™ 2000 transfection reagent following the instructions provided by the manufacturer. The sequence of the shRNA and siRNA were listed in the Table S2 (Supporting Information)

### Immunofluorescence Staining

The kidney sections were dewaxed, rehydrated, and rinsed with deionized water. Antigen retrieval was then performed by microwaving, followed by blocking for 1 hour. Next, the sections were incubated with primary antibodies, including Lgals3, α‐SMA, Collagen1, PKM2, L‐lactyllysine, and H3K18la. All sections were counterstained with DAPI. Fluorescence images were captured using a laser scanning confocal microscope.

The HK‐2 cells were seeded onto dish and fixed with 3.7% paraformaldehyde for 15 min. Then cells were permeabilized, washed and blocked with 5% goat serum for 2 h. Cells were incubated with antibodies against Lgals3, α‐SMA, Collagen1, PKM2, Trim21 and H3K18la at 4 °C overnight. Fluorochrome‐conjugated secondary antibodies were dropped onto slices in the dark. The cell nuclei were counterstained with DAPI. Images were acquired using a confocal microscopy.

### Immunoblot Analysis

Cells or tissues were lysed with RIPA supplemented with protease and phosphatase inhibitor. Subsequently, the proteins were deposited onto a polyvinylidene fluoride (PVDF) membrane (Millipore) blocked using 5% milk. The membranes were exposed to primary antibodies for 12h and subsequently exposed to secondary antibodies. The ECL kit was utilized to detect the target protein band. The primary antibodies were as followers: anti‐Lgals3 (CST, cat#87 985, 1:1000), anti‐Fibronectin (Abcam, cat#ab2413, 1:1000), anti‐α‐SMA (ProteinTech Group, cat#14395‐1‐AP, 1:2000), anti‐Collagen1 (Abcam, cat#ab138492, 1:1000), anti‐PKM2 (CST, cat#4053, 1:1000), anti‐lacty‐lysine (PTM BioLab, cat# 1401RM, 1:1000), anti‐H3K9la (PTM BioLab, cat# 1419RM, 1:1000); anti‐H3K14la (PTM BioLab, cat# 1414RM, 1:1000); anti‐H3K18la (PTM BioLab, cat# 1406RM, 1:1000); anti‐H3K56la (PTM BioLab, cat# 1421RM, 1:1000); anti‐H3 (CST, cat#4499, 1:2000); anti‐H4K5la (PTM BioLab, cat# 1407RM, 1:1000); anti‐H4K8la (PTM BioLab, cat# 1415RM, 1:1000); anti‐H4K12la (PTM BioLab, cat# 1411RM, 1:1000); anti‐FGFR4 (abclonal, cat#A7555, 1:1000) and anti‐GAPDH (CST, cat#2118, 1:1000).

### Quantitative Real‐Time PCR (RT‐qPCR)

RNA was extracted from cultured cells following the manufacturer's protocol. One microgram of RNA was reverse transcribed into cDNA using a gDNA eraser. Subsequently, 2 µL of cDNA was combined with qPCR SYBR Master Mix, forward and reverse primers, and milliQ water to reach a final volume of 20 µL. Real‐time PCR was conducted using SYBR Green Master Mix on an Applied Biosystems StepOne Real‐Time PCR System, following a standard cycling protocol. The different primers were listed in Table S3 (Supporting Information).

### Measurement of Extracellular Acidification Rate (ECAR)

The extracellular acidification rate (ECAR) was measured using the Seahorse XFe 96 Extracellular Flux Analyzer (Agilent Biotechnologies). Briefly, cells were seeded into 24‐well plates and washed with Seahorse detection buffer. The analyzer then administered Rot/AA and 2‐DG (2‐deoxy‐glucose) to determine the glycolytic proton efflux rate, basal glycolysis rate, and compensatory glycolysis rate.

### Glycolytic Process Evaluation

Cells were incubated with 2‐[N‐(7‐nitrobenz‐2‐oxa‐1,3‐diazol‐4‐yl)amino]‐2‐deoxy‐D‐glucose (2‐NBDG) at a concentration of 10 µM for 1 hour and subsequently washed with PBS. Fluorescence intensity was then measured using flow cytometry with excitation wavelengths of 488 nm and 542 nm.

### Measurement of Lactate Levels

Lactate levels were evaluated in cell culture medium from HK‐2 cells by using the colorimetric lactate assay kit following the manufacturer's instructions.

### Measurement of ROS

ROS generation was assessed using the ROS detection kit (Thermo Fisher, USA) following the manufacturer's specifications. Semi‐quantification of ROS levels was evaluated using ImageJ software.

### Co‐IP and Ubiquitination Assays

The cells were lysed with IP lysis buffer after required specific transfection time. The samples were incubated with 2–5 µg antibodies (IgG/PKM2/FLAG/His/Myc) overnight at 4 °C. Protein A/G beads were then added and incubated at 4 °C overnight. The beads were washed three times with elution buffer and resuspended with SDS loading buffer before being heated at 95 °C for 10 min, and then Western blot analysis was performed.

For ubiquitination analysis, Lysates were precipitated with anti‐His or PKM2 antibody, and the IP procedure was repeated as described above. Immunoblotting was performed with anti‐HA or Ub antibody to measure ubiquitination levels.

### IP‐MS

The beads samples were subjected to incubation in a reaction solution consisting of 1% sodium deoxycholate, 100 mM Tris‐HCl (pH 8.5), 10 mM tris(2‐carboxyethyl) phosphine, and 40 mM chloroacetamide. The incubation was carried out at a temperature of 95 °C for a duration of 10 minutes. The eluates were diluted with an equivalent volume of water and then underwent trypsin digestion overnight. Trypsin was added at a ratio of 1:50 for digestion at 37 °C. Following centrifugation at a force of 12 000 times the acceleration due to gravity for a duration of 15 minutes, the peptide was purified using desalting columns that were produced in‐house using SDB. The eluate was subjected to vacuum evaporation and thereafter kept at a temperature of ‐20 °C for future utilization. The samples were analyzed using an UltiMate 3000 RSLCnano system that was connected to a Q Exactive HF mass spectrometer via a Nanospray Flex ion source.

### CUT‐Tag

The CUT&Tag assay was performed using the Hyperactive In‐Situ ChIP Library Prep Kit for Illumina, following the manufacturer's instructions. In brief, both wild‐type (WT) HK2 cells and Lgals3‐knockdown HK‐2 cells were targeted using ConA beads. The cell membranes were permeabilized with the nonionic detergent digitonin. Subsequently, the cells were incubated with H3K18la antibody (PTM‐1427RM, PTM BIO, Hangzhou, China), a secondary antibody, and Hyperactive pA‐Tn5 Transposase. The resulting purified PCR products were evaluated with the Agilent 2100 Bioanalyzer. Finally, these libraries were sequenced on the Illumina NovaSeq6000 platform, producing 150 bp paired‐end reads for further analysis.

### Chip‐qPCR

Cells were initially crosslinked by incubating with 2 mM disuccinimidyl glutarate for 45 minutes, followed by 1% formaldehyde for 10 minutes. The chromatin was then immunoprecipitated using the specified antibodies at 4 °C overnight with continuous rotation. Subsequently, the DNA was captured, eluted, reverse‐crosslinked, and purified. Finally, real‐time PCR amplification was performed using specific primers listed in Table S3 (Supporting Information).

### Statistical Analysis

Statistical analysis was performed using GraphPad Prism 8.0. For comparisons between two groups, an independent‐sample Student's t‐test was employed. To compare three or more experimental groups, one‐way and two‐way analyses of variance (ANOVA) were utilized. A p‐value of less than 0.05 was deemed statistically significant.

## Conflict of Interest

The authors declare no conflict of interest.

## Author Contributions

Z.Y., Y.S., S.Y. were co‐first authors. X.Z., and F.C. contributed equally to this work. Z.Y. performed conceptualisation, validation. Y.S. wrote – review & edited the original draft. S.Y. performed investigation. L.L. performed software. B.L. performed formal analysis. T.Y. performed data curation. Y.X. performed supervision. L.C. performed data curation, investigation. W.Y. performed data curation. W.L., F.C., and X.Z. performed supervision, funding acquisition.

## Supporting information



Supporting Information

## Data Availability

The data that support the findings of this study are available from the corresponding author upon reasonable request.
