# Supplementary material for: Lgals3 Promotes Calcium Oxalate Crystal Formation and Kidney Injury Through Histone Lactylation‐Mediated FGFR4 Activation
Source: Adv Sci (Weinh). 2025 Feb 4;12(12):2413937. doi: 10.1002/advs.202413937 (PMC11947994; doi:10.1002/advs.202413937)
Supplement: Supplementary file 1 — Supporting Information [file ADVS-12-2413937-s001.docx]

**Supproting Information**

**Lgals3 promotes Calcium oxalate crystal formation and kidney injury through histone lactylation-mediated FGFR4 activation**

Zehua Ye, Yushi Sun, Songyuan Yang, Lei Li, Bojun Li, Yuqi Xia, Tianhui Yuan, Weimin Yu, Lijia Chen, Xiangjun Zhou*, Fan Cheng*


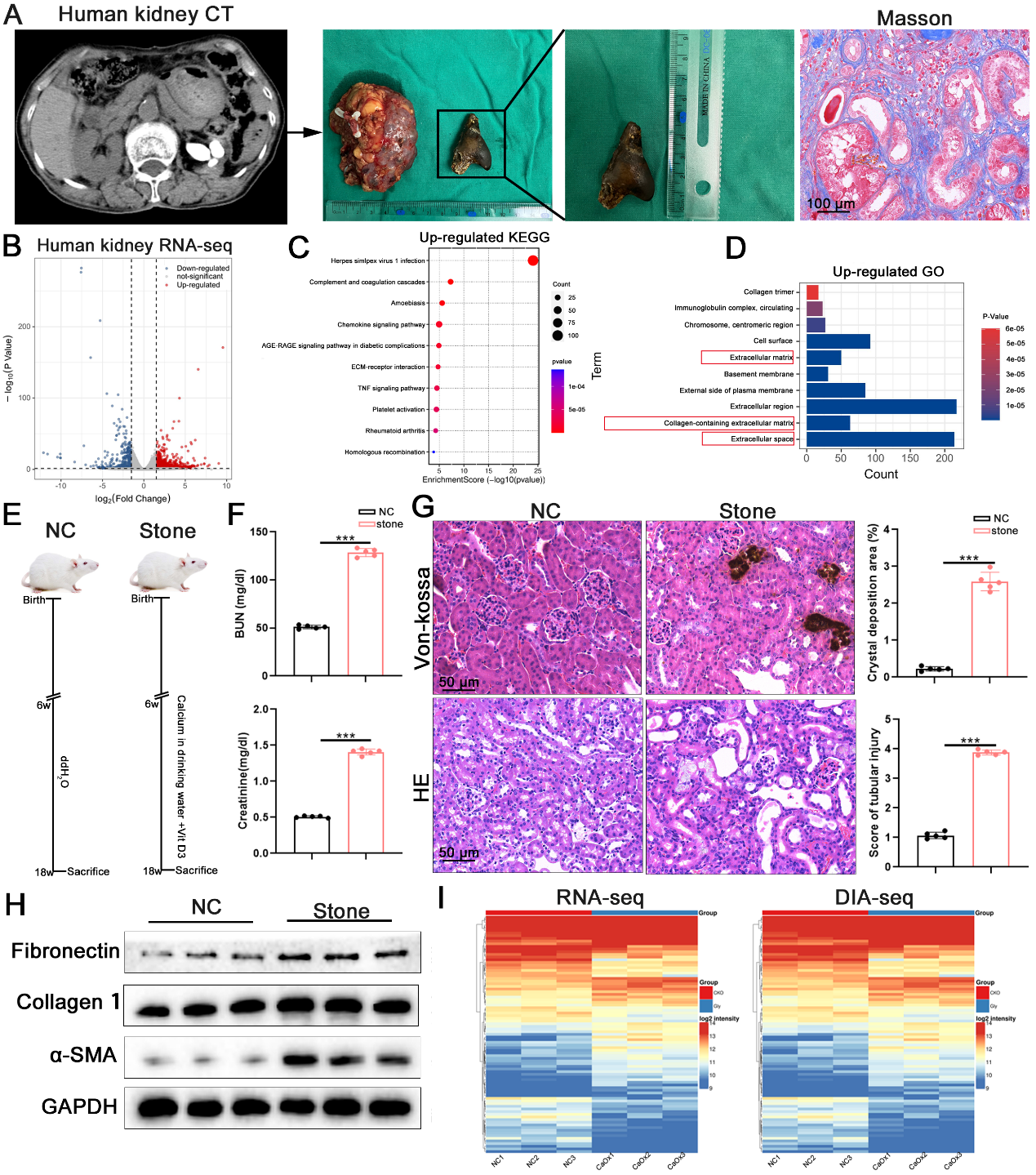


**Figure S1 The multi-omics were performed in the kidney of CaOx stone.**

**A)** The patients with CaOx kidney stone and the Masson staining of kidney. **B)** Volcano plot for differentially expressed genes (DEGs) in the kidney tissues from control group and kidney stone patient group. Non-tumor renal tissues from patients with renal cell carcinoma were used as control. **C)** Bubble chart showing the Kyoto Encyclopedia of Genes and Genomes (KEGG) pathway enrichment analysis of the up-regulated genes in CaOx group. **D)** Bubble chart showing the Gene Ontology (GO) enrichment analysis of the up-regulated genes in CaOx group. **E)** The schematic of the experimental design. **F)** The BUN and Scr level in blood from NC mice and CaOx stone mice (n = 5 mice per group). **G)** Representative images and quantification of HE and Von-kossa staining in kidney tissues from NC mice and CaOx stone mice (scale bar = 50 μm, n = 5 mice per group). **H)** Immunoblots of the protein expression levels and quantification of Fibronectin, α-SMA and Collagen1 in kidney tissues from NC mice and CaOx stone mice (n = 5 mice per group). **I)** The RNA-seq and DIA-proteomic were performed in the kidney tissues from control group and kidney stone group mice. ***P*<0.01, compared to the NC group.


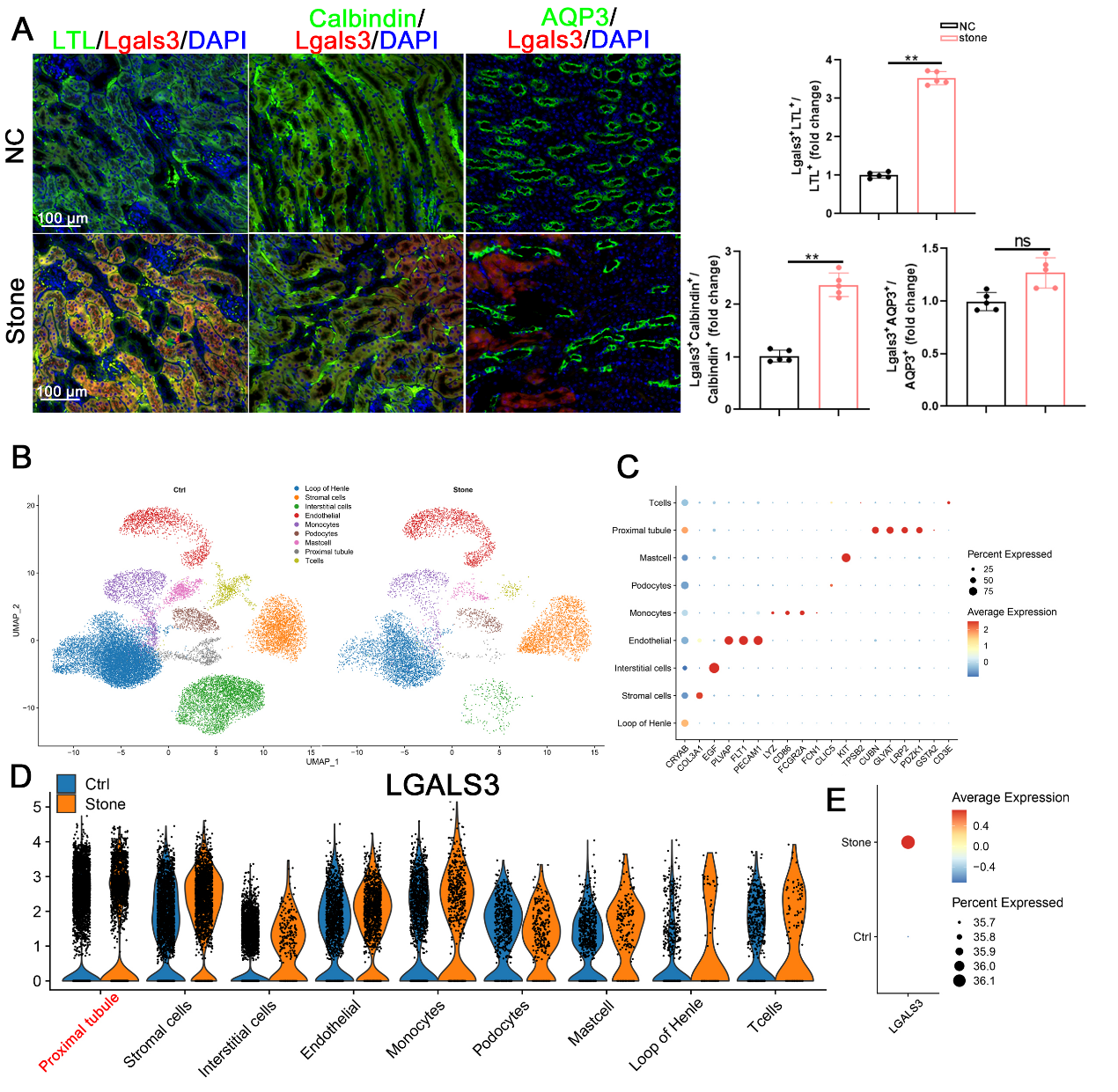


**FigureS2 The expression of Lgals3 in kidney tissues and online single database.**

**A)** The immunofluorescence images and quantification of Lgals3 with different tubule markers in the kidney tissues from NC mice and CaOx stone mice (scale bar = 100 μm, n = 5 mice per group). **B-C)** The cell was divided into 9 different types according marker gene based on the CaOx kidney stone patient single-cell sequence database (gse231569). **D-E)** The expression of Lgals3 in different cell types. ***P*<0.01, compared to the NC group.


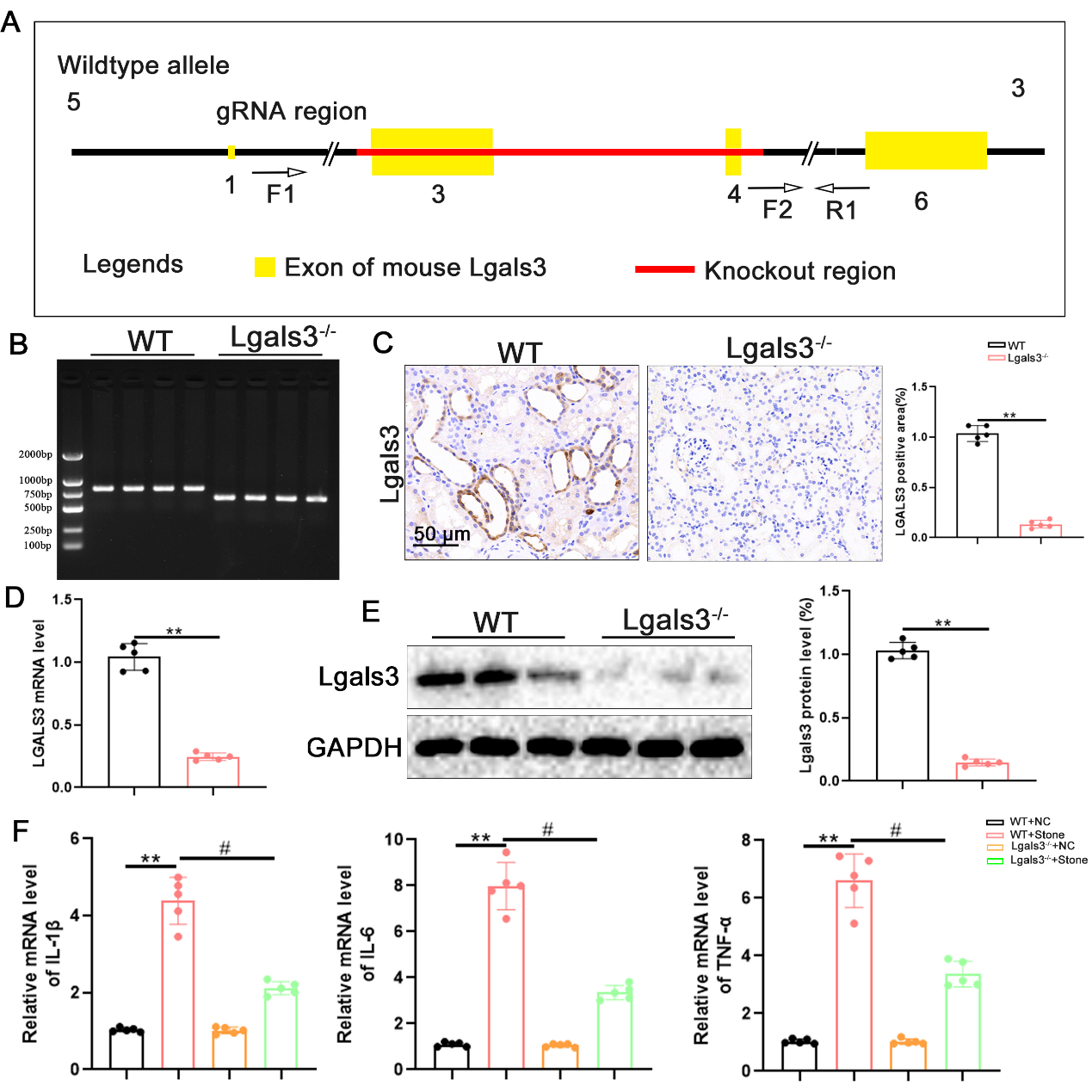


**FigureS3 The establishment of Lgals3 knockout mice.**

**A)** The schedule of Lgals3 knockout mice. **B)** The Lgals3 knockout mice genotyping was confirmed by tail preparation and PCR (n = 5 mice per group). **C)** Representative image and quantification of the immunohistochemical staining of Lgals3 in kidney tissues from WT mice and Lgals3 knockout (Lgals3^-/-^) mice (scale bar = 50 μm, n = 5 mice per group). **D-E)** The mRNA and protein level of Lgals3 in kidney tissues from WT mice and Lgals3 knockout (Lgals3^-/-^) mice (n = 5 mice per group). ***P*<0.01, compared to the WT mice group. **F)** The mRNA level of IL-1β, IL-6 and TNF-α in kidney tissues from WT mice and Lgals3 knockout (Lgals3^-/-^) mice (n = 5 mice per group). ***P*<0.01, compared to the NC group; ^#^*P*<0.05, compared with WT-Stone mice.


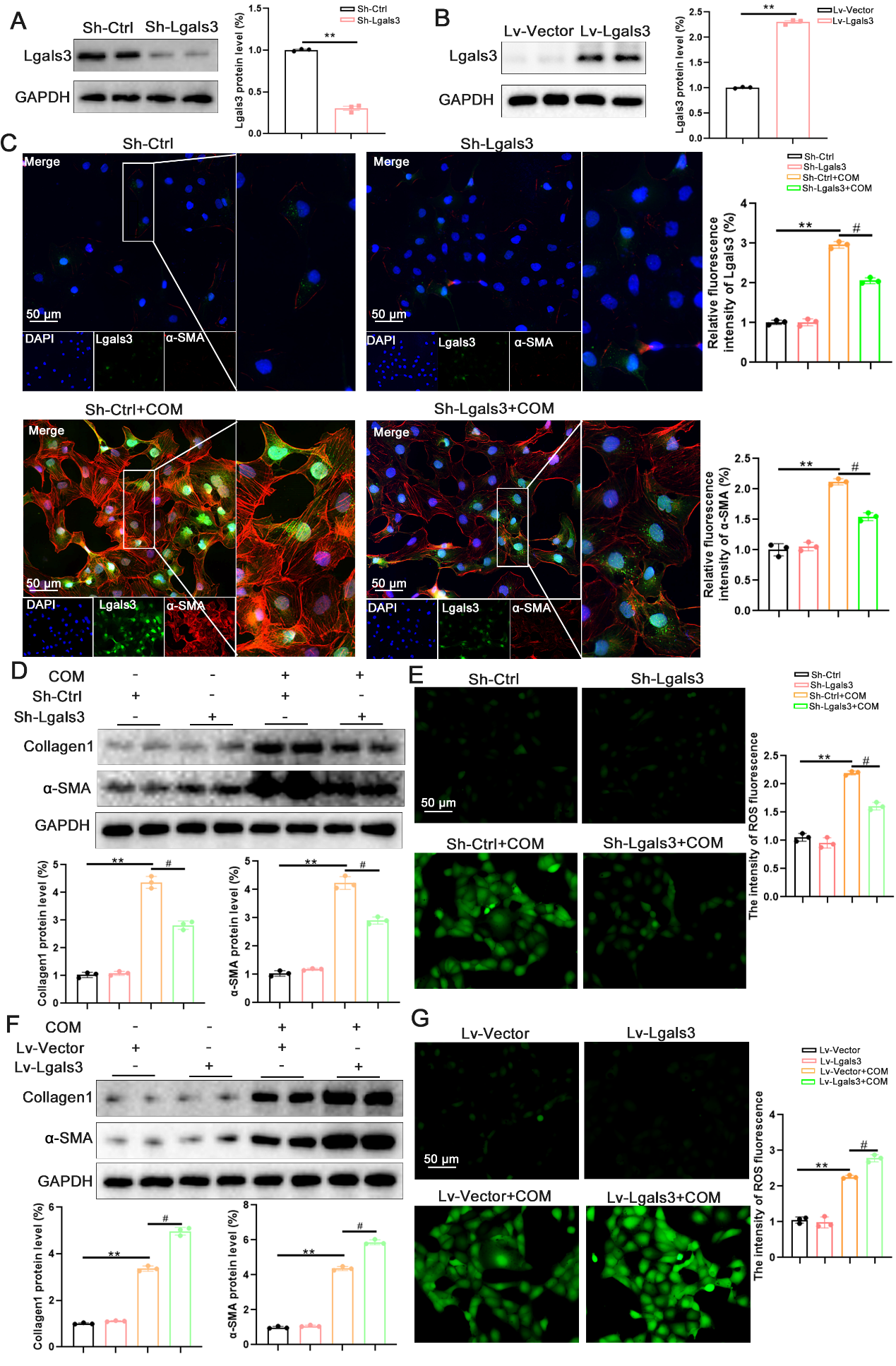


**FigureS4 Lgals3 deficiency or overexpression inhibits or promotes CaOx crystal formation and renal fibrosis *in vitro***

**A)** Immunoblots of the protein expression levels and quantification of Lgals3 in HK-2 cell from Sh-Ctrl group and Sh-Lgals3 group (n = 3 per group). ***P*<0.01, compared to the Sh-Ctrl group. **B)** Immunoblots of the protein expression levels and quantification of Lgals3 in HK-2 cell from Lv-Vector group and Lv-Lgals3 group (n = 3 per group). ***P*<0.01, compared to the Lv-Vector group. **C)** Representative images of the immunofluorescence staining of Lgals3 (green) and α-SMA (red) in Lgals3 knockdown HK-2 cells with COM treated with 48h (n = 3 per group). **D)** Immunoblots of the protein expression levels and quantification of Collagen1 and α-SMA in Lgals3 knockdown HK-2 cells with COM treated for 48h (n = 3 per group). **E)** Representative images of the immunofluorescence staining of ROS in Lgals3 knockdown HK-2 cells with COM treated with 48h (n = 3 per group). ***P*<0.01, compared to the Sh-Ctrl group; ^#^*P*<0.05, compared with Sh-Ctrl+COM group. **F)** Immunoblots of the protein expression levels and quantification of Collagen1 and α-SMA in Lgals3 overexpression HK-2 cells with COM treated for 48h (n = 3 per group). **G)** Representative images of the immunofluorescence staining of ROS in Lgals3 overexpression HK-2 cells with COM treated for 48h (n = 3 per group). ***P*<0.01, compared to the Lv-Vector group; ^#^*P*<0.05, compared with Lv-Vector +COM group.


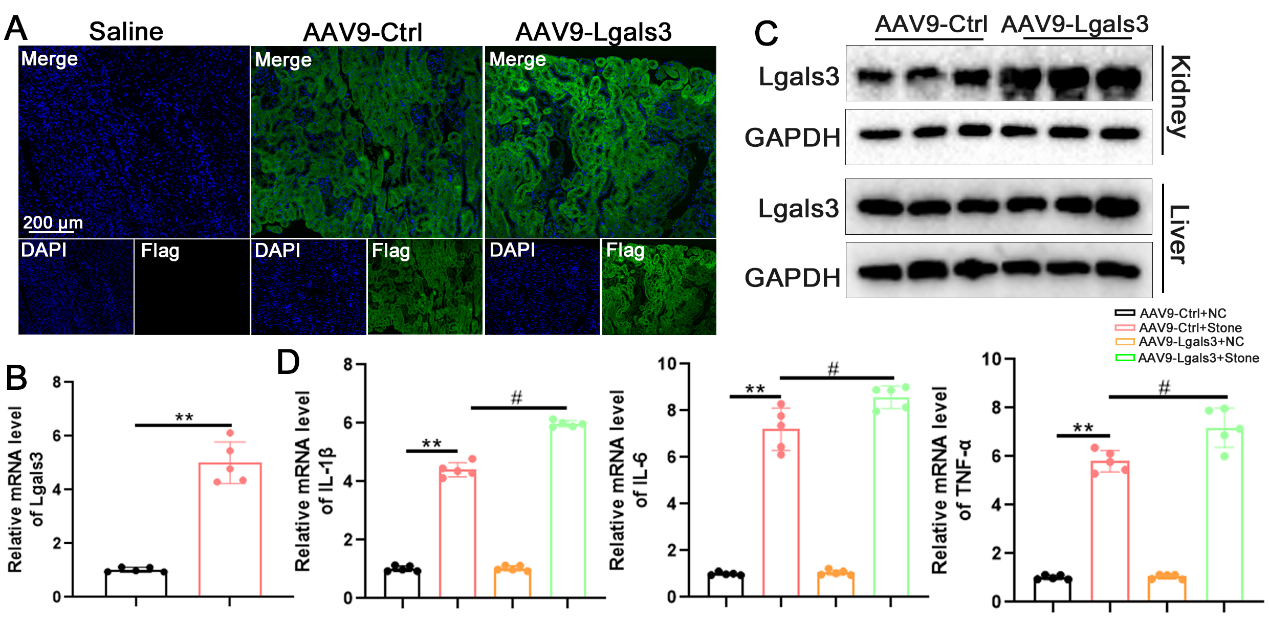


**FigureS5 The overexpression of Lgals3 *in vivo***

**A)** The success of AAV9 transduction was determined by fluorescence detection. (scale bar = 200 um, n = 5 mice per group). **B-C)** The mRNA and protein level of Lgals3 in kidney tissues from AAV9-NC mice and AAV9-Lgals3 mice (n = 5 mice per group). ***P*<0.01, compared to the AAV9-Ctrl group; **D)** The mRNA level of IL-1β, IL-6 and TNF-α in kidney tissues from WT mice and Lgals3 knockout (Lgals3^-/-^) mice (n = 5 mice per group). ***P*<0.01, compared to the NC group; ^#^*P*<0.05, compared with AAV9-Ctrl-Stone mice.


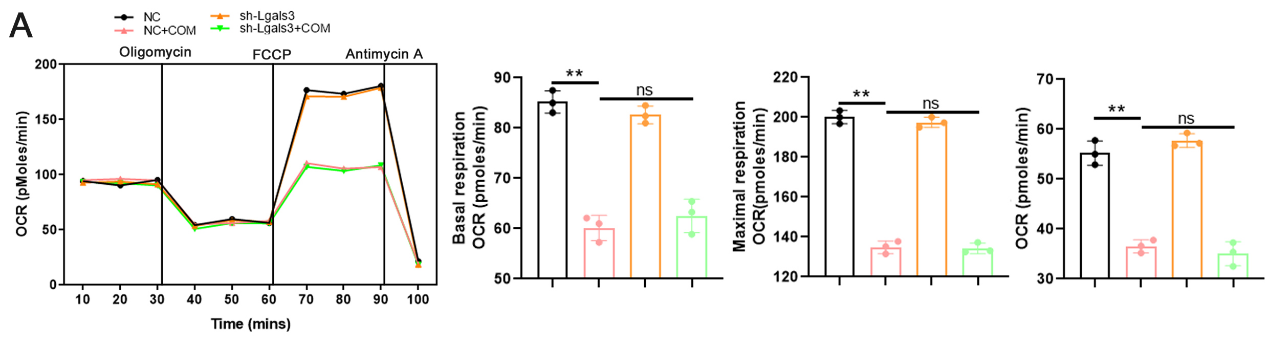


**FigureS6 The effect of Lgals3 on oxidative phosphorylation.**

**A)** Mitochondrial respiration profiles of Lgals3 knockdown and WT HK-2 cells in the presence or absence of COM as determined by the Seahorse analyzer, n=3 per group. ***P*<0.01, compared to the NC group;


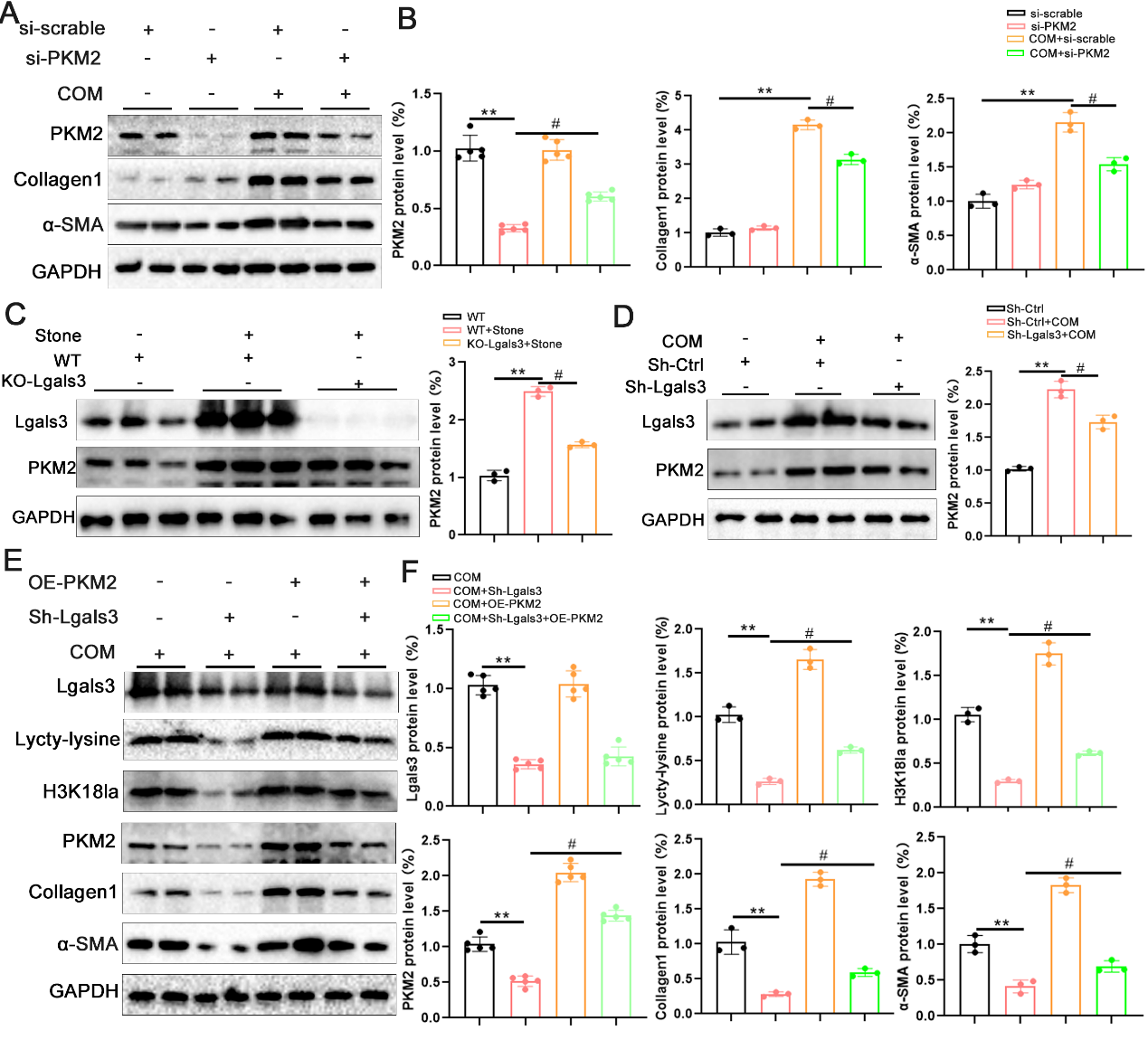


**FigureS7 Lgals3 regulates PKM2 expression in the process of CaOx stone formation.**

**A-B)** Immunoblots of the protein expression levels and quantification of Collagen1 and α-SMA in PKM2 knockdown HK-2 cells with COM treated for 48h (n = 3 per group). ***P*<0.01, compared to the si-scrable group; ^#^*P*<0.05, compared with si-scrable +COM group. **C)** Immunoblots of the protein expression levels and quantification of PKM2 in kidney tissues from WT mice and Lgals3 knockout (Lgals3^-/-^) mice (n = 5 mice per group). ***P*<0.01, compared to the WT group; ^#^*P*<0.05, compared with WT+Stone group. **D)** Immunoblots of the protein expression levels and quantification of PKM2 in Lgals3 knockdown HK-2 cells with COM treated for 48h (n = 3 per group). ***P*<0.01, compared to the Sh-Ctrl group; ^#^*P*<0.05, compared with Sh-Ctrl+COM group. **E-F)** Immunoblots of the protein expression levels and quantification of Lgals3, Lycty-lysine, H3K18la, PKM2, Collagen1 and α-SMA in PKM2 overexpression HK-2 cells with COM treated for 48h (n = 3 per group). ***P*<0.01, compared to the COM group; ^#^*P*<0.05, compared with Sh-Lgals3+COM group.


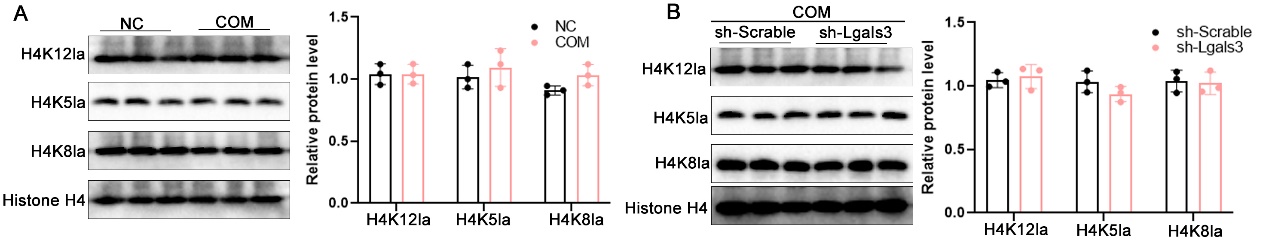


**FigureS8 The expression of various lactylation sites on histone H4**

**A)** Immunoblots of the protein expression levels and quantification of H4K12la, H4K5la, and H4K8la in HK-2 cells with COM treated for 48h (n = 3 per group). **B)** Immunoblots of the protein expression levels and quantification of H4K12la, H4K5la, and H4K8la in Lgals3 knockdown HK-2 cells with COM treated for 48h (n = 3 per group).


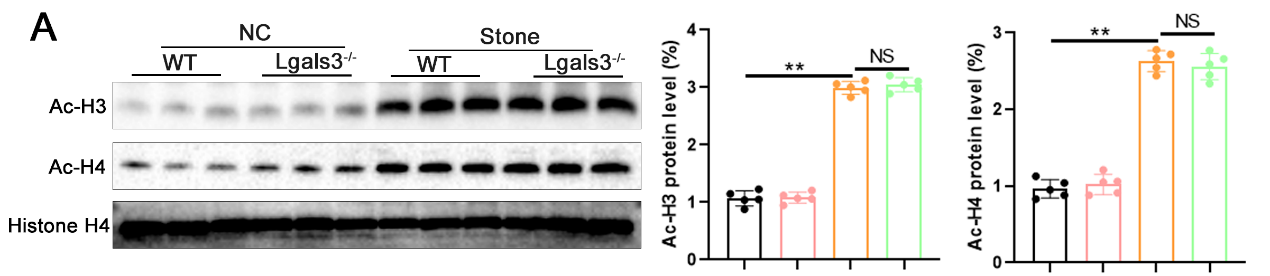


**FigureS9 The expression of acetylation histone H3 and histone H4**

**A)** Immunoblots of the protein expression levels and quantification of acetylation histone H3 and histone H4 in kidney tissues from WT mice and Lgals3 knockout (Lgals3-/-) mice (n = 5 mice per group). ***P*<0.01, compared to the NC group.


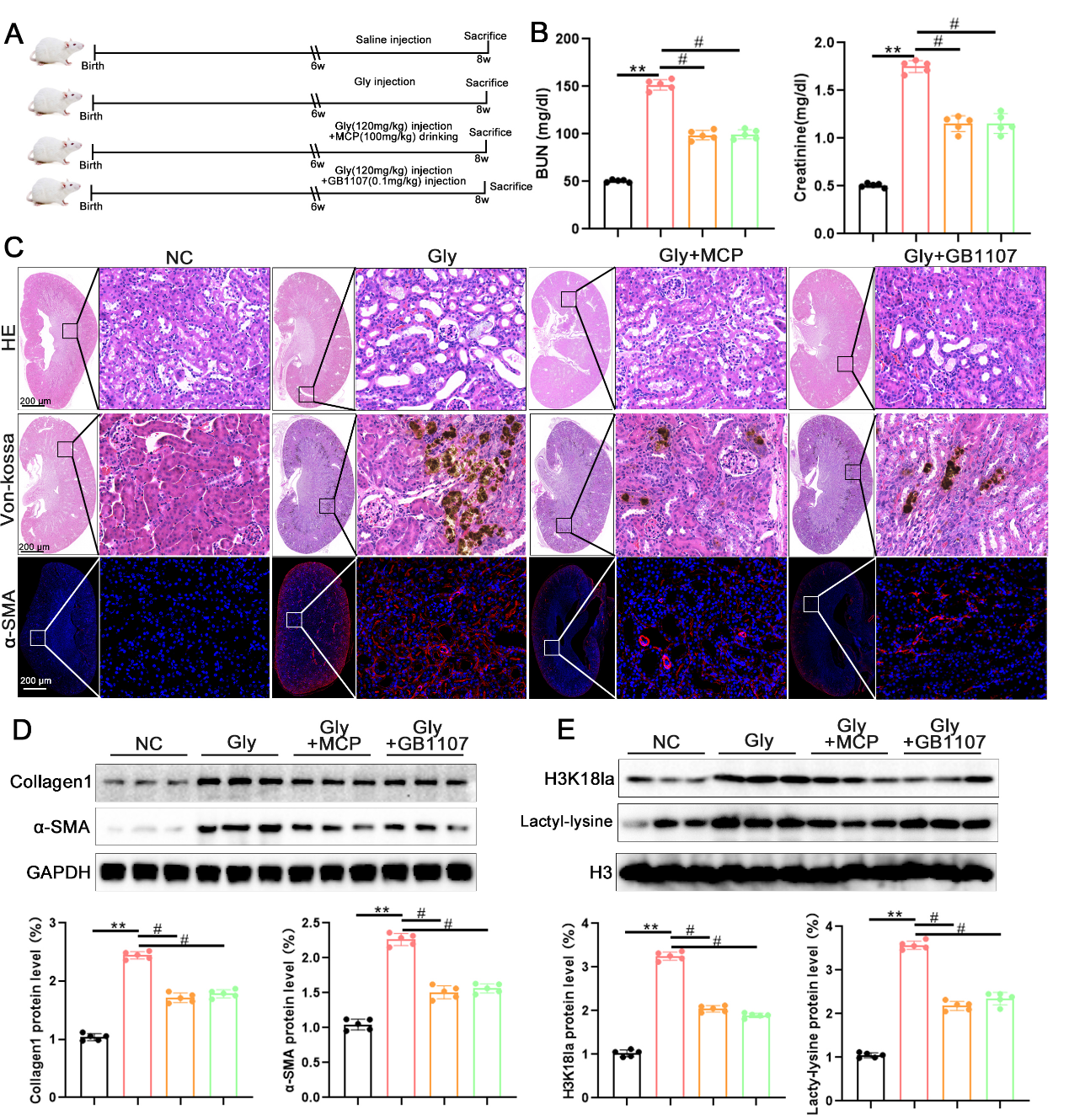


**FigureS10 Pharmacological inhibition of Lgals3 ameliorates CaOx crystal formation and renal fibrosis**

**A)** The schematic of the experimental design. **B)** The BUN and Scr level in blood from CaOx stone+MCP-1 mice and CaOx stone +GB1107 mice (n = 5 mice per group). **C)** Representative images and quantification of HE, Von-kossa and α-SMA staining in kidney tissues from CaOx stone+MCP-1 mice and CaOx stone +GB1107 mice (scale bar = 50 um, n = 5 mice per group). **D-E)** Immunoblots of the protein expression levels and quantification of α-SMA, Collagen1, H3K18la and Lacty-lysine in kidney tissues from CaOx stone+MCP-1 mice and CaOx stone +GB1107 mice (n = 5 mice per group). ***P*<0.01, compared to the NC group; ^#^*P*<0.05, compared with Gly group.

**Table S1: The clinical characteristics of patients**

| Sample number | Gender | Age [ys] | eGFR | Diagnosis | stone composition |
| --- | --- | --- | --- | --- | --- |
| 1 | female | 67 | 86.55 | RCC | - |
| 2 | female | 54 | 78.05 | RCC | - |
| 3 | male | 58 | 82.33 | RCC | - |
| 4 | male | 63 | 78.7 | RCC | - |
| 5 | male | 68 | 73.54 | RCC | - |
| 6 | female | 59 | 76.65 | RCC | - |
| 7 | male | 62 | 82.21 | RCC | - |
| 8 | female | 57 | 81.56 | RCC | - |
| 9 | female | 58 | 75.64 | RCC | - |
| 10 | male | 51 | 79.23 | RCC | - |
| 11 | male | 54 | 26.32 | Kieney stone; non-function kidney | uric acid stones |
| 12 | female | 61 | 31.2 | Kieney stone; non-function kidney | uric acid stones |
| 13 | female | 53 | 15.78 | Kieney stone; non-function kidney | struvite stones |
| 14 | female | 43 | 17.23 | Kieney stone; non-function kidney | struvite stones |
| 15 | male | 56 | 19.33 | Kieney stone; non-function kidney | COM stones |
| 16 | female | 52 | 24.56 | Kieney stone; non-function kidney | COM stones |
| 17 | male | 59 | 28.71 | Kieney stone; non-function kidney | COM stones |
| 18 | male | 42 | 16.5 | Kieney stone; non-function kidney | COM stones |
| 19 | female | 51 | 31.2 | Kieney stone; non-function kidney | COM stones |
| 20 | male | 44 | 28.6 | Kieney stone; non-function kidney | COM stones |

**Table S2**

siRNA/shRNA

| Description | Sense |
| --- | --- |
| Lgals3-shRNA | GCAGTACAATCATCGGGTTAA |
| PKM2-siRNA | GCUCCAUACGCCAUUCGUTT |

**Table S3**

Primers

| Gene | Forward (5 to 3 sequence) | Reverse (5 to 3 sequence) |
| --- | --- | --- |
| Lgals1 | AGTCTTCTGACAGCTGGTGC | CGAAGGCACTCTCCAGGTTT |
| Lgals2 | GCGAATCCACCATTGTCTGC | GGCTGAAGCACAGGTGATCT |
| Lgals3 | GTCCGGAGCCAGCCAAC | AGGCCATCCTTGAGGGTTTG |
| Lgals4 | TCAACTTCATCGGAGGCCAG | GCTGTTCAGCTGTTGATGGC |
| Lgals5 | ACCCGAACCTAGCTGTACCT | AAAGCAATGTCACCCCCACA |
| Lgals6 | CTTCTTCGGAGTCCAACCTGT | CCACATATGGCAGACACGGA |
| Lgals7 | TGAGAATTCGCGGCTTGGTT | CTGTTGAAGACCACCTCCGA |
| PKM2 | CCTGATAGCTCGTGAGGCTG | GTGGAGTGACTTGAGGCTCG |
| FGFR4 | TTCCGGCAAGTCAAGCTCAT | TTCCCAAGCACCAGCGAG |
| IL-1β | TGCCACCTTTTGACAGTGATG | TGATGTGCTGCTGCGAGATT |
| IL-6 | GACAAAGCCAGAGTCCTTCAGA | TGTGACTCCAGCTTATCTCTTGG |
| TNF-α | GATCGGTCCCCAAAGGGATG | CCACTTGGTGGTTTGTGAGTG |
| GAPDH | GGAGCGAGATCCCTCCAAAAT | GGCTGTTGTCATACTTCTCATGG |
| FGFR4  Chip | CCGAGCAGGCAGTAAGTC | ACGACAGAGTGGTAGGAAACAG |
